# Supplementary material for: Giant orbital magnetoelectric effect and current-induced magnetization switching in twisted bilayer graphene
Source: Nat Commun. 2020 Apr 3;11:1650. doi: 10.1038/s41467-020-15473-9 (PMC7125167; doi:10.1038/s41467-020-15473-9)
Supplement: Supplementary file 1 — Supplementary Information [file 41467_2020_15473_MOESM1_ESM.pdf]

Supplementary Information for

Giant Orbital Magneto-electric effect and Current-driven Magnetization Switching in Twisted Bilayer Graphene

He *et al.*

SUPPLEMENTARY NOTE 1: CONTINUUM MODEL FOR THE TWISTED BILAYER GRAPHENE WITH HETEROSTRAIN

In the monolayer graphene, we set the primitive lattice vector as

$$\mathbf{a}_1 = \sqrt{3}\left(\frac{1}{2}, \frac{\sqrt{3}}{2}\right) d, \qquad \mathbf{a}_2 = \sqrt{3}\left(-\frac{1}{2}, \frac{\sqrt{3}}{2}\right) d. \tag{1}$$

The vectors which link the origin of the unit cell to the respective sublattices $\alpha = A, B$ are $\boldsymbol{\delta}_A = \mathbf{0}, \boldsymbol{\delta}_B = d\,(0,1)$. The corresponding reciprocal primitive lattice vectors are:

$$\mathbf{b}_1 = \frac{4\pi}{3d}\left(\frac{\sqrt{3}}{2}, \frac{1}{2}\right), \qquad \mathbf{b}_2 = \frac{4\pi}{3d}\left(-\frac{\sqrt{3}}{2}, \frac{1}{2}\right). \tag{2}$$

The Dirac points are located at the Brillouin zone corners $\mathbf{K}_\pm = \frac{4\pi}{3d}\left(\frac{\sqrt{3}}{2}, \frac{1}{2}\right)$. The uniaxial strain will deform the honeycomb lattice and change the shape of both the real and reciprocal lattices. The Dirac points in the reciprocal space will be shifted by strain. The uniaxial strain tensor $\boldsymbol{\mathcal{E}}$ can be written as

$$\boldsymbol{\mathcal{E}} = \varepsilon \begin{pmatrix} -\cos^2\phi + \nu\sin^2\phi & -(1+\nu)\cos\phi\sin\phi \\ -(1+\nu)\cos\phi\sin\phi & \nu\cos^2\phi - \sin^2\phi \end{pmatrix}, \tag{3}$$

with ϕ denoting the direction of the applied uniaxial strain and $\nu = 0.165$ the Poison's ratio for the graphene. The uniaxial strain transforms the coordinates in the real and reciprocal spaces as

$$\tilde{\mathbf{r}} = (1 + \boldsymbol{\mathcal{E}})\,\mathbf{r}, \qquad \tilde{\mathbf{k}} = \left(1 - \boldsymbol{\mathcal{E}}^{\mathrm{T}}\right)\mathbf{k}. \tag{4}$$

As a result, the strain changes the position of the Dirac points in the reciprocal space to

$$\tilde{\mathbf{K}}_\xi = \left(1 - \boldsymbol{\mathcal{E}}^{\mathrm{T}}\right)\mathbf{K}_\xi - \xi\mathbf{A}. \tag{5}$$

Here, $\xi = \pm 1$ is the valley index and the effective gauge field can be written as

$$\mathbf{A} = \frac{\beta}{d}\left(\mathcal{E}_{xx} - \mathcal{E}_{yy}, -2\mathcal{E}_{xy}\right), \tag{6}$$

with $\beta = 1.57$ and $d = 1.42\mathring{A}$. The bottom layer is coupled with the aligned boron nitride substrate and the top layer is not affected. The Hamiltonian for the bottom layer graphene at valley ξ reads

$$\tilde{\mathcal{H}}_{\mathrm{b}} = \sum_{\mathbf{q},s,\xi} a^\dagger_{\mathrm{b},s,\xi}(\mathbf{q})\, h_{\mathrm{b},\xi}(\mathbf{q})\, a_{\mathrm{b},s,\xi}(\mathbf{q}) = \sum_{\mathbf{q},s,\xi} a^\dagger_{\mathrm{b},s,\xi}(\mathbf{q}) \left[\xi\hbar v_{\mathrm{F}}\hat{\mathbf{R}}_{-\frac{\theta}{2}}\left(1 + \boldsymbol{\mathcal{E}}^{\mathrm{T}}\right)(\mathbf{q} + \xi\mathbf{A})\cdot\boldsymbol{\sigma} + \Delta\sigma_z\right] a_{\mathrm{b},s,\xi}(\mathbf{q}). \tag{7}$$

Here, $\hat{\mathbf{R}}_{-\frac{\theta}{2}} = \cos\frac{\theta}{2} + i\sigma_y\sin\frac{\theta}{2}$ is the rotation matrix, the momentum is denoted as $\mathbf{q} = \mathbf{k} - \left(1 - \boldsymbol{\mathcal{E}}^{\mathrm{T}}\right)\mathbf{K}_\xi$, and $a^{(\dagger)}_{s,\xi}(\mathbf{q})$ is a two component creation (annihilation) operator with valley index ξ and spin index $s = \uparrow, \downarrow$. The top layer graphene has the Hamiltonian

$$\mathcal{H}_{\mathrm{t}} = \sum_{\mathbf{q},s,\xi} a^\dagger_{\mathrm{t},s,\xi}(\mathbf{q})\, h_{\mathrm{t},\xi}(\mathbf{q})\, a_{\mathrm{t},s,\xi}(\mathbf{q}) = \sum_{\mathbf{q},s,\xi} a^\dagger_{\mathrm{t},s,\xi}(\mathbf{q})\, \xi\hbar v_{\mathrm{F}}\hat{\mathbf{R}}_{\frac{\theta}{2}}\mathbf{q}\cdot\boldsymbol{\sigma} a_{\mathrm{t},s,\xi}(\mathbf{q}). \tag{8}$$

Then we denote the tunneling matrix element from the bottom layer to the top layer as [1–3]

$$\begin{aligned} \tilde{T}^{\alpha,\beta}_{\hat{\mathbf{R}}_{-\frac{\theta}{2}}\tilde{\mathbf{K}}_\xi+\mathbf{q},\hat{\mathbf{R}}_{\frac{\theta}{2}}\mathbf{K}_\xi+\mathbf{q}'} =& \frac{1}{3}t_\perp \Big[\delta_{\hat{\mathbf{R}}_{-\frac{\theta}{2}}\tilde{\mathbf{K}}_\xi+\mathbf{q},\hat{\mathbf{R}}_{\frac{\theta}{2}}\mathbf{K}_\xi+\mathbf{q}'} + e^{i\tilde{\mathbf{b}}_2\cdot\left(\tilde{\boldsymbol{\delta}}_\alpha - \tilde{\boldsymbol{\delta}}_\beta\right)}\delta_{\hat{R}_{-\frac{\theta}{2}}\left(\tilde{\mathbf{K}}_\xi+\tilde{\mathbf{b}}_2\right)+\mathbf{q},\hat{\mathbf{R}}_{\frac{\theta}{2}}(\mathbf{K}_\xi+\mathbf{b}_2)+\mathbf{q}'} \\ &+ e^{-i\tilde{\mathbf{b}}_1\cdot\left(\tilde{\boldsymbol{\delta}}_\alpha - \tilde{\boldsymbol{\delta}}_\beta\right)}\delta_{\hat{\mathbf{R}}_{-\frac{\theta}{2}}\left(\tilde{\mathbf{K}}_\xi-\tilde{\mathbf{b}}_1\right)+\mathbf{q},\hat{\mathbf{R}}_{\frac{\theta}{2}}(\mathbf{K}_\xi-\mathbf{b}_1)+\mathbf{q}'}\Big], \end{aligned} \tag{9}$$

so the strain deformed interlayer Hamiltonian can be written as

$$\tilde{\mathcal{H}}_{\mathrm{int}} = \sum_{\mathbf{q},s,\xi} a^\dagger_{\mathrm{b},s,\xi}(\mathbf{q}) \left[\tilde{T}_{\xi\tilde{\mathbf{q}}_{\mathrm{b}}}\delta_{\mathbf{q}'-\mathbf{q},\xi\tilde{\mathbf{q}}_{\mathrm{b}}} + \tilde{T}_{\xi\tilde{\mathbf{q}}_{\mathrm{tr}}}\delta_{\mathbf{q}'-\mathbf{q},\xi\tilde{\mathbf{q}}_{\mathrm{tr}}} + \tilde{T}_{\xi\tilde{\mathbf{q}}_{\mathrm{tl}}}\delta_{\mathbf{q}'-\mathbf{q},\xi\tilde{\mathbf{q}}_{\mathrm{tl}}}\right] a_{\mathrm{t},s,\xi}(\mathbf{q}') + h.c., \tag{10}$$

where

$$\tilde{T}_{\xi\tilde{\mathbf{q}}_{\mathrm{b}}} = \frac{1}{3}t_{\perp}\begin{pmatrix}1 & 1\\ 1 & 1\end{pmatrix}, \tag{11}$$

$$\tilde{T}_{\xi\tilde{\mathbf{q}}_{\mathrm{tr}}} = \frac{1}{3}t_{\perp}\begin{pmatrix}1 & e^{-i\xi\frac{2\pi}{3}\left(1+\sqrt{3}\mathcal{E}_{xx}\mathcal{E}_{xy}+\sqrt{3}\mathcal{E}_{xy}\mathcal{E}_{yy}-\mathcal{E}_{xy}^{2}-\mathcal{E}_{yy}^{2}\right)}\\ e^{i\xi\frac{2\pi}{3}\left(1+\sqrt{3}\mathcal{E}_{xx}\mathcal{E}_{xy}+\sqrt{3}\mathcal{E}_{xy}\mathcal{E}_{yy}-\mathcal{E}_{xy}^{2}-\mathcal{E}_{yy}^{2}\right)} & 1\end{pmatrix}, \tag{12}$$

$$\tilde{T}_{\xi\tilde{\mathbf{q}}_{\mathrm{tl}}} = \frac{1}{3}t_{\perp}\begin{pmatrix}1 & e^{i\xi\frac{2\pi}{3}\left(1-\sqrt{3}\mathcal{E}_{xx}\mathcal{E}_{xy}-\sqrt{3}\mathcal{E}_{xy}\mathcal{E}_{yy}-\mathcal{E}_{xy}^{2}-\mathcal{E}_{yy}^{2}\right)}\\ e^{-i\xi\frac{2\pi}{3}\left(1-\sqrt{3}\mathcal{E}_{xx}\mathcal{E}_{xy}-\sqrt{3}\mathcal{E}_{xy}\mathcal{E}_{yy}-\mathcal{E}_{xy}^{2}-\mathcal{E}_{yy}^{2}\right)} & 1\end{pmatrix}, \tag{13}$$

with

$$\begin{aligned}\tilde{\mathbf{q}}_{\mathrm{b}} =& \hat{\mathbf{R}}_{-\frac{\theta}{2}}\left(1-\boldsymbol{\mathcal{E}}^{\mathrm{T}}\right)\mathbf{K}_{+} - \hat{\mathbf{R}}_{\frac{\theta}{2}}\mathbf{K}_{+}\\ =& -\frac{4\pi}{3\sqrt{3}d}\left(\mathcal{E}_{xx}\cos\tfrac{\theta}{2}+\mathcal{E}_{xy}\sin\tfrac{\theta}{2},\ \left(2-\mathcal{E}_{xx}\right)\sin\tfrac{\theta}{2}+\mathcal{E}_{xy}\cos\tfrac{\theta}{2}\right)\end{aligned} \tag{14}$$

$$\begin{aligned}\tilde{\mathbf{q}}_{\mathrm{tr}} =& \hat{\mathbf{R}}_{-\frac{\theta}{2}}\left(1-\boldsymbol{\mathcal{E}}^{\mathrm{T}}\right)\left(\mathbf{K}_{+}+\mathbf{b}_{2}\right) - \hat{\mathbf{R}}_{\frac{\theta}{2}}\left(\mathbf{K}_{+}+\mathbf{b}_{2}\right)\\ =& \frac{2\pi}{9d}\left(\left(\sqrt{3}\mathcal{E}_{xx}-3\mathcal{E}_{xy}\right)\cos\tfrac{\theta}{2}+\left(6+\sqrt{3}\mathcal{E}_{xy}-3\mathcal{E}_{yy}\right)\sin\tfrac{\theta}{2},\ -\left(3\mathcal{E}_{yy}-\sqrt{3}\mathcal{E}_{xy}\right)\cos\tfrac{\theta}{2}+\left(2\sqrt{3}+3\mathcal{E}_{xy}-\sqrt{3}\mathcal{E}_{xx}\right)\sin\tfrac{\theta}{2}\right)\end{aligned} \tag{15}$$

$$\begin{aligned}\tilde{\mathbf{q}}_{\mathrm{tl}} =& \hat{\mathbf{R}}_{-\frac{\theta}{2}}\left(1-\boldsymbol{\mathcal{E}}^{\mathrm{T}}\right)\left(\mathbf{K}_{+}-\mathbf{b}_{1}\right) - \hat{\mathbf{R}}_{\frac{\theta}{2}}\left(\mathbf{K}_{+}-\mathbf{b}_{1}\right)\\ =& \frac{2\pi}{9d}\left(\left(\sqrt{3}\mathcal{E}_{xx}+3\mathcal{E}_{xy}\right)\cos\tfrac{\theta}{2}-\left(6-\sqrt{3}\mathcal{E}_{xy}-3\mathcal{E}_{xx}\right)\sin\tfrac{\theta}{2},\ \left(3\mathcal{E}_{yy}+\sqrt{3}\mathcal{E}_{xy}\right)\cos\tfrac{\theta}{2}+\left(2\sqrt{3}-3\mathcal{E}_{xy}-\sqrt{3}\mathcal{E}_{xx}\right)\sin\tfrac{\theta}{2}\right).\end{aligned} \tag{16}$$

The interlayer hopping strength is taken as $t_{\perp} = 0.33$eV. Finally, the Hamiltonian for the twisted bilayer graphene aligned with boron nitride substrate is written as

$$\begin{aligned}\mathcal{H} =& \tilde{\mathcal{H}}_{\mathrm{b}} + \mathcal{H}_{\mathrm{t}} + \tilde{\mathcal{H}}_{\mathrm{int}}\\ =& \sum_{\mathbf{q},s,\xi} A_{s,\xi}^{\dagger}\left(\mathbf{q}\right)h_{\xi}\left(\mathbf{q}\right)A_{s,\xi}\left(\mathbf{q}\right),\end{aligned} \tag{17}$$

where $A_{s,\xi}\left(\mathbf{q}\right)$ has infinite number of components representing the series of states $a_{\mathrm{b},s,\xi}\left(\mathbf{q}\right)$, $a_{\mathrm{t},s,\xi}\left(\mathbf{q}'\right)$ with $\mathbf{q}-\mathbf{q}' = \xi\mathbf{q}_{\mathrm{b}}, \xi\mathbf{q}_{\mathrm{tr}}, \xi\mathbf{q}_{\mathrm{tl}}$. The Hamiltonian matrix $h_{\xi}\left(\mathbf{q}\right)$ in the truncated basis $\left[a_{\mathrm{b},s,\xi}\left(\mathbf{q}\right), a_{\mathrm{t},s,\xi}\left(\mathbf{q}+\xi\mathbf{q}_{\mathrm{b}}\right), a_{\mathrm{t},s,\xi}\left(\mathbf{q}+\xi\mathbf{q}_{\mathrm{tr}}\right), a_{\mathrm{t},s,\xi}\left(\mathbf{q}+\xi\mathbf{q}_{tl}\right)\right]^{\mathrm{T}}$ then has the form

$$h_{\xi}\left(\mathbf{q}\right) = \begin{pmatrix}h_{\mathrm{b},\xi}\left(\mathbf{q}\right) & \tilde{T}_{\xi\tilde{\mathbf{q}}_{\mathrm{b}}} & \tilde{T}_{\xi\tilde{\mathbf{q}}_{\mathrm{tr}}} & \tilde{T}_{\xi\tilde{\mathbf{q}}_{\mathrm{tl}}}\\ \tilde{T}_{\xi\tilde{\mathbf{q}}_{\mathrm{b}}}^{\dagger} & h_{\mathrm{t},\xi}\left(\mathbf{q}+\xi\tilde{\mathbf{q}}_{\mathrm{b}}\right) & 0 & 0\\ \tilde{T}_{\xi\tilde{\mathbf{q}}_{\mathrm{tr}}}^{\dagger} & 0 & h_{\mathrm{t},\xi}\left(\mathbf{q}+\xi\tilde{\mathbf{q}}_{\mathrm{tr}}\right) & 0\\ \tilde{T}_{\xi\tilde{\mathbf{q}}_{\mathrm{tl}}}^{\dagger} & 0 & 0 & h_{\mathrm{t},\xi}\left(\mathbf{q}+\xi\tilde{\mathbf{q}}_{\mathrm{tl}}\right)\end{pmatrix}. \tag{18}$$

We consider 42 sites in the hexagonal reciprocal lattice so that $h_{\xi}\left(\mathbf{q}\right)$ is a 84×84 matrix in the calculation. In Supplementary Fig. 1, the orbital magnetic moment distribution in the mini-Brillouin zone for a sample with strain is presented. The uniaxial strain along the zigzag direction of the bottom layer with $\varepsilon = 0.2\%, \varepsilon = 0.3\$, \varepsilon = 0.5\%$ is used. The orbital magnetic moments reach larger values with the increase of the uniaxial strain and can even reach $100\mu_{\mathrm{b}}$. The corresponding magnetoelectric susceptibility for $\varepsilon = 0.2\%, \varepsilon = 0.3\$, \varepsilon = 0.5\%$ is shown in Supplementary Fig. 2. It can be seen that the electric field induced orbital magnetization gets enhanced as the uniaxial strain increases. At $\varepsilon = 0.5\%$, the maximal orbital magnetization can exceed $0.04\mu_{\mathrm{b}}\mathrm{nm}^{-2}$. In the presence of the time reversal symmetry, the Hamiltonian matrix $h_{\xi}\left(\mathbf{q}\right)$ satisfies the relation

$$h_{+}\left(\mathbf{q}\right) = h_{-}^{*}\left(-\mathbf{q}\right), \tag{19}$$

and the energy eigenvalues satisfy the relation

$$E_{s,+,\nu}\left(\mathbf{q}\right) = E_{s,-,\nu}\left(-\mathbf{q}\right). \tag{20}$$

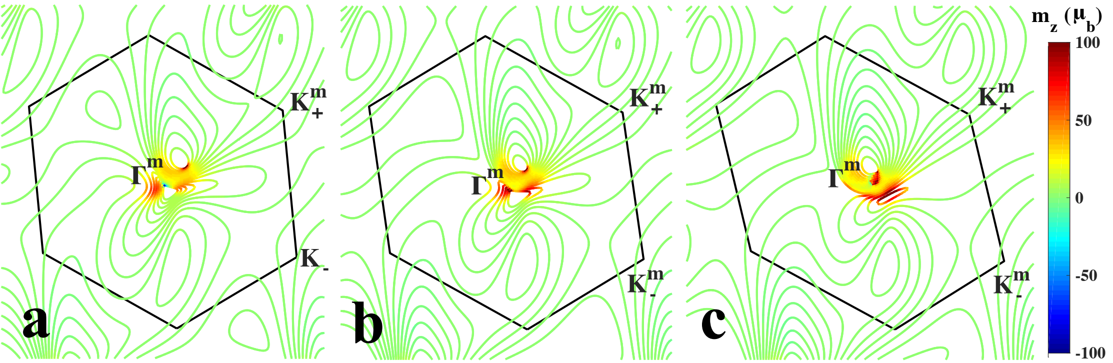

Supplementary Fig. 1: The orbital magnetic moment at different conduction band energy contours in the mini-Brillouin zone. The heterostrain we considered is along the zigzag direction of the bottom layer graphene with $\varepsilon = 0.2\%$ for (a), $\varepsilon = 0.3\%$ for (b) and $\varepsilon = 0.5\%$ for (c).

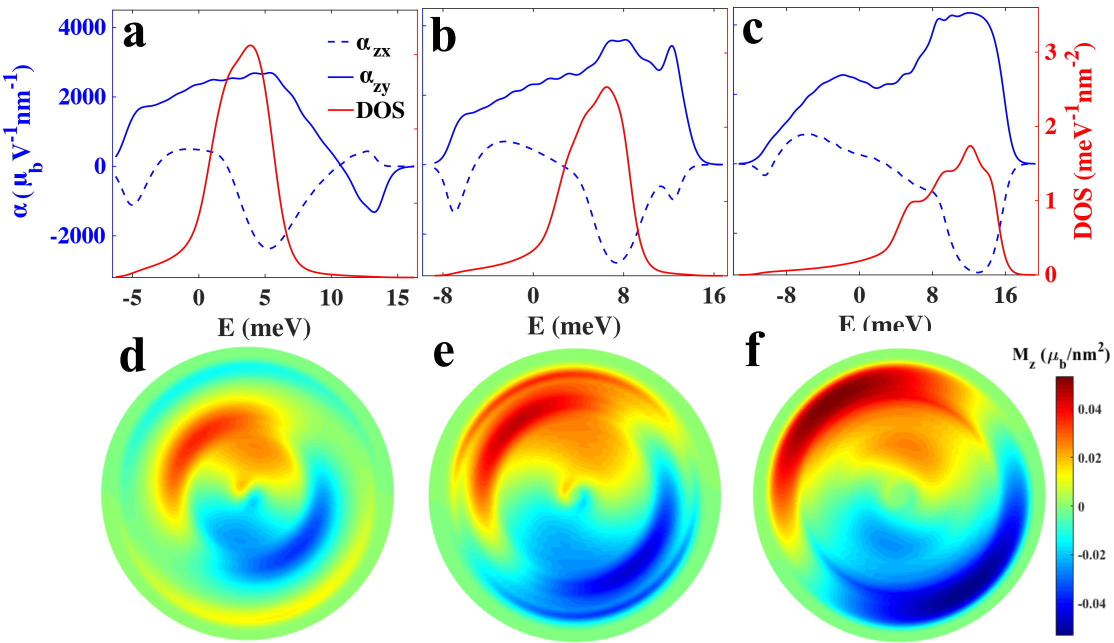

Supplementary Fig. 2: The magnetoelectric susceptibility α_{zx}, α_{zy} at $\varepsilon = 0.2\%$ for (a), $\varepsilon = 0.3\%$ for (b), and $\varepsilon = 0.5\%$ for (c). The resultant orbital magnetization in the presence of electric field $E = 10^4$V/m is correspondingly present in (d), (e), (f). The twisting angle is fixed to be $\theta = 1.2°$.

[1] J. M. B. L. dos Santos, N. M. R. Peres, and A. H. C. Neto, *Graphene Bilayer with a Twist: Electronic Structure*, Phys. Rev. Lett. **99**, 256802 (2007).

[2] J. M. B. L. dos Santos, N. M. R. Peres, and A. H. C. Neto, *Continuum Model of the Twisted Graphene Bilayer*, Phys. Rev. B **86**, 155449 (2012).

[3] R. Bistritzer and A. H. MacDonald, *Moiré bands in twisted double-layer graphene*, Proc. Natl. Acad. Sci. U.S.A. **108**, 12233 (2011).
