# Supplementary material for: Giant orbital magnetoelectric effect and current-induced magnetization switching in twisted bilayer graphene
Source: Nat Commun. 2020 Apr 3;11:1650. doi: 10.1038/s41467-020-15473-9 (PMC7125167; doi:10.1038/s41467-020-15473-9)
Supplement: Supplementary file 3 — Description of Additional Supplementary Files [file 41467_2020_15473_MOESM3_ESM.pdf]

Description of Additional Supplementary Files

Supplementary Data 1

The Supplementary Data 1 file includes all the Matlab codes for plotting all the four figures in the main text.
