# Supplementary material for: Giant orbital magnetoelectric effect and current-induced magnetization switching in twisted bilayer graphene
Source: Nat Commun. 2020 Apr 3;11:1650. doi: 10.1038/s41467-020-15473-9 (PMC7125167; doi:10.1038/s41467-020-15473-9)
Supplement: Supplementary file 4 — Supplementary Data 1 [file 41467_2020_15473_MOESM4_ESM.pdf]

Matlab codes for plotting all the four figures in the maintext.

Band structure plotting

```
theta=1.2/180*pi;
t=2.8;
MM1=1*0.017;
MM2=-0;
d=1.42;
hbar_vf=3/2*d*t;
t_vert=0.33;
epsilon=1*1e-3;
phi=-0*theta/2+0*pi/5;
nu=0.165;
Epsilon=epsilon*[cos(phi),-sin(phi);sin(phi),cos(phi)]*[-1,0;0,nu]*[c
os(phi),sin(phi);-sin(phi),cos(phi)];
EExx=Epsilon(1,1);
EEyy=Epsilon(2,2);
EExy=Epsilon(1,2);
beta=1.57;
A=beta/d*[EExx-EEyy,-2*EExy];

qb0=8*pi*sin(1/2*theta)/(3*sqrt(3)*d)*[0,-1];
qtr0=8*pi*sin(1/2*theta)/(3*sqrt(3)*d)*[sqrt(3)/2,1/2];
qtl0=8*pi*sin(1/2*theta)/(3*sqrt(3)*d)*[-sqrt(3)/2,1/2];
b1m0=8*pi*sin(1/2*theta)/(3*d)*[1/2,-sqrt(3)/2];
b2m0=8*pi*sin(1/2*theta)/(3*d)*[1/2,sqrt(3)/2];

NN=5;
[NN1,NN2]=meshgrid(-NN:NN);
NN1=reshape(NN1,(2*NN+1)^2,1);
NN2=reshape(NN2,(2*NN+1)^2,1);
K_0=NN1*b1m0+NN2*b2m0;
K_1=K_0-ones((2*NN+1)^2,1)*qb0;
K_2=K_0+ones((2*NN+1)^2,1)*qb0;
NNK1=find(abs(K_1(:,1)+i*K_1(:,2))<=0.8*NN*max(norm(b1m0),norm(b2m0))
);
NNK2=find(abs(K_2(:,1)+i*K_2(:,2))<=0.8*NN*max(norm(b1m0),norm(b2m0))
);

TKqb=t_vert/3*[1,1;1,1];
TKqtr=t_vert/3*[1,exp(-i*2*pi/3*(1+sqrt(3)*EExx*EExy+sqrt(3)*EEyy*EEx
y-EExy^2-EEyy^2));
```

```
exp(i*2*pi/3*(1+sqrt(3)*EExx*EExy+sqrt(3)*EEyy*EExy-EExy^2-EEyy^2)),1
];
TKqtl=t_vert/3*[1,exp(i*2*pi/3*(1-sqrt(3)*EExx*EExy-sqrt(3)*EEyy*EExy
-EExy^2-EEyy^2));

exp(-i*2*pi/3*(1-sqrt(3)*EExx*EExy-sqrt(3)*EEyy*EExy-EExy^2-EEyy^2)),
1];
T_Kqb=t_vert/3*[1,1;1,1];
T_Kqtr=t_vert/3*[1,exp(i*2*pi/3*(1+sqrt(3)*EExx*EExy+sqrt(3)*EEyy*EEx
y-EExy^2-EEyy^2));

exp(-i*2*pi/3*(1+sqrt(3)*EExx*EExy+sqrt(3)*EEyy*EExy-EExy^2-EEyy^2)),
1];
T_Kqtl=t_vert/3*[1,exp(-i*2*pi/3*(1-sqrt(3)*EExx*EExy-sqrt(3)*EEyy*EE
xy-EExy^2-EEyy^2));

exp(i*2*pi/3*(1-sqrt(3)*EExx*EExy-sqrt(3)*EEyy*EExy-EExy^2-EEyy^2)),1
];
qb=-4*pi/(3*sqrt(3)*d)*[EExx*cos(theta/2)+EExy*sin(theta/2),(2-EExx)*
sin(theta/2)+EExy*cos(theta/2)];
qtr=2*pi/(9*d)*[(sqrt(3)*EExx-3*EExy)*cos(theta/2)+(6-3*EEyy+sqrt(3)*
EExy)*sin(theta/2),-(3*EEyy-sqrt(3)*EExy)*cos(theta/2)+(2*sqrt(3)-sqr
t(3)*EExx+3*EExy)*sin(theta/2)];
qtl=2*pi/(9*d)*[(sqrt(3)*EExx+3*EExy)*cos(theta/2)-(6-3*EEyy-sqrt(3)*
EExy)*sin(theta/2),(3*EEyy+sqrt(3)*EExy)*cos(theta/2)+(2*sqrt(3)-sqrt
(3)*EExx-3*EExy)*sin(theta/2)];
b1m=2*pi/(3*d)*[-(sqrt(3)*EExx+EExy)*cos(theta/2)+(2-EEyy-sqrt(3)*EEx
y)*sin(theta/2),-(EEyy+sqrt(3)*EExy)*cos(theta/2)-(2*sqrt(3)-sqrt(3)*
EExx-EExy)*sin(theta/2)];
b2m=2*pi/(3*d)*[(sqrt(3)*EExx-EExy)*cos(theta/2)+(2-EEyy+sqrt(3)*EExy
)*sin(theta/2),-(EEyy-sqrt(3)*EExy)*cos(theta/2)+(2*sqrt(3)-sqrt(3)*E
Exx+EExy)*sin(theta/2)];
ZZ=zeros(2);

[NN1,NN2]=meshgrid(-NN:NN);
NN1=reshape(NN1,(2*NN+1)^2,1);
NN2=reshape(NN2,(2*NN+1)^2,1);
K0=NN1*b1m+NN2*b2m;
K1=K0-ones((2*NN+1)^2,1)*qb;
K2=K0+ones((2*NN+1)^2,1)*qb;
K1=K1(NNK1,:);
K2=K2(NNK2,:);
NK1=length(K1);
NK2=length(K2);
```

```
kx1=linspace(qtr(1),qtr(1)+qb(1),50);
ky1=linspace(qtr(2),qtr(2)+qb(2),50);
kx2=linspace(qtr(1)+qb(1),0,50);
ky2=linspace(qtr(2)+qb(2),0,50);
kx3=linspace(0,qtr(1)+1/2*qb(1),50);
ky3=linspace(0,qtr(2)+1/2*qb(2),50);
kx4=linspace(qtr(1)+1/2*qb(1),qtr(1),50);
ky4=linspace(qtr(2)+1/2*qb(2),qtr(2),50);
kx=[kx1,kx2,kx3,kx4];
ky=[ky1,ky2,ky3,ky4];

for n1=1:length(K1)
    for n2=1:length(K2)
        if norm((K1(n1,:)-K2(n2,:))+qb)<1/2*norm(qb)
            HK_T(2*n1-1:2*n1,2*n2-1:2*n2)=TKqb;
            H_K_T(2*n1-1:2*n1,2*n2-1:2*n2)=T_Kqb';
        elseif norm((K1(n1,:)-K2(n2,:))-qb)<1/2*norm(qb)
            HK_T(2*n1-1:2*n1,2*n2-1:2*n2)=TKqb';
            H_K_T(2*n1-1:2*n1,2*n2-1:2*n2)=T_Kqb;
        elseif norm((K1(n1,:)-K2(n2,:))+qtr)<1/2*norm(qtr)
            HK_T(2*n1-1:2*n1,2*n2-1:2*n2)=TKqtr;
            H_K_T(2*n1-1:2*n1,2*n2-1:2*n2)=T_Kqtr';
        elseif norm((K1(n1,:)-K2(n2,:))-qtr)<1/2*norm(qtr)
            HK_T(2*n1-1:2*n1,2*n2-1:2*n2)=TKqtr';
            H_K_T(2*n1-1:2*n1,2*n2-1:2*n2)=T_Kqtr;
        elseif norm((K1(n1,:)-K2(n2,:))+qtl)<1/2*norm(qtl)
            HK_T(2*n1-1:2*n1,2*n2-1:2*n2)=TKqtl;
            H_K_T(2*n1-1:2*n1,2*n2-1:2*n2)=T_Kqtl';
        elseif norm((K1(n1,:)-K2(n2,:))-qtl)<1/2*norm(qtl)
            HK_T(2*n1-1:2*n1,2*n2-1:2*n2)=TKqtl';
            H_K_T(2*n1-1:2*n1,2*n2-1:2*n2)=T_Kqtl;
        else
            HK_T(2*n1-1:2*n1,2*n2-1:2*n2)=ZZ;
            H_K_T(2*n1-1:2*n1,2*n2-1:2*n2)=ZZ;
        end
    end
end
for n=1:length(kx)
    q=[kx(n),ky(n)];
    for n1=1:length(K1)

qK1=((1+EExx)*(q(1)-K1(n1,1)+A(1))+EExy*(q(2)-K1(n1,2)+A(2)))+i*(EExy
*(q(1)-K1(n1,1)+A(1))+(1+EEyy)*(q(2)-K1(n1,2)+A(2)));
```

```
        HK1(2*n1-1:2*n1,2*n1-1:2*n1)=hbar_vf*[0, 
conj(qK1)*exp(i*theta/2); 
            qK1*exp(-i*theta/2), 0]+MM1*[1,0;0,-1];
        q_K2=q(1)-K1(n1,1)+i*(q(2)-K1(n1,2));
        H_K2(2*n1-1:2*n1,2*n1-1:2*n1)=-hbar_vf*[0, q_K2*exp(i*theta/2); 
            conj(q_K2)*exp(-i*theta/2), 0]+MM2*[1,0;0,-1];
    end
    for n1=1:length(K2)
        qK2=q(1)-K2(n1,1)+i*(q(2)-K2(n1,2));
        HK2(2*n1-1:2*n1,2*n1-1:2*n1)=hbar_vf*[0, 
conj(qK2)*exp(-i*theta/2); 
            qK2*exp(i*theta/2), 0]+MM2*[1,0;0,-1];

q_K1=((1+EExx)*(q(1)-K2(n1,1)-A(1))+EExy*(q(2)-K2(n1,2)-A(2)))+i*(EEx
y*(q(1)-K2(n1,1)-A(1))+(1+EEyy)*(q(2)-K2(n1,2)-A(2)));
        H_K1(2*n1-1:2*n1,2*n1-1:2*n1)=-hbar_vf*[0, 
q_K1*exp(-i*theta/2); 
            conj(q_K1)*exp(i*theta/2), 0]+MM1*[1,0;0,-1];
    end
    HK=[HK1,HK_T;HK_T',HK2];
    H_K=[H_K2,H_K_T;H_K_T',H_K1];
    EK(:,n)=eig(HK);
    E_K(:,n)=eig(H_K);
    n
end
figure
plot(linspace(0,4,length(kx)),EK(NK1+NK2-1,:)*1e3,'c-',linspace(0,4,l
ength(kx)),EK(NK1+NK2,:)*1e3,'b-',linspace(0,4,length(kx)),EK(NK1+NK2
+1,:)*1e3,'r-',linspace(0,4,length(kx)),EK(NK1+NK2+2,:)*1e3,'m-','Lin
eWidth',2),colormap(jet)
grid on,axis([0,4,-20,20])
set(gca,'XTick',[0,1,2,3,4])
set(gca,'YTick',[-20,-10,0,10,20])
set(gca,'XTickLabel',{'K^m','K^m_-','\Gamma^m','M^m','K^m'})
set(gca,'FontName','Times New Roman','FontSize', 28,'fontweight','b')
ylabel('E (meV)','FontName','Times New 
Roman','FontSize',39,'fontweight','b')
%hold on%figure
%plot(linspace(0,4,length(kx)),E_K,'--'),grid 
on,axis([0,4,-0.02,0.02])

theta=1.2/180*pi;
t=2.8;
MM1=1*0.017;
```

```
MM2=-0;
d=1.42;
hbar_vf=3/2*d*t;
t_vert=0.33;
epsilon=0*1e-3;
phi=-theta/2+0*pi/5;
nu=0.165;
Epsilon=epsilon*[cos(phi),-sin(phi);sin(phi),cos(phi)]*[-1,0;0,nu]*[c
os(phi),sin(phi);-sin(phi),cos(phi)];
EExx=Epsilon(1,1);
EEyy=Epsilon(2,2);
EExy=Epsilon(1,2);
beta=1.57;
A=beta/d*[EExx-EEyy,-2*EExy];

qb0=8*pi*sin(1/2*theta)/(3*sqrt(3)*d)*[0,-1];
qtr0=8*pi*sin(1/2*theta)/(3*sqrt(3)*d)*[sqrt(3)/2,1/2];
qtl0=8*pi*sin(1/2*theta)/(3*sqrt(3)*d)*[-sqrt(3)/2,1/2];
b1m0=8*pi*sin(1/2*theta)/(3*d)*[1/2,-sqrt(3)/2];
b2m0=8*pi*sin(1/2*theta)/(3*d)*[1/2,sqrt(3)/2];

NN=5;
[NN1,NN2]=meshgrid(-NN:NN);
NN1=reshape(NN1,(2*NN+1)^2,1);
NN2=reshape(NN2,(2*NN+1)^2,1);
K_0=NN1*b1m0+NN2*b2m0;
K_1=K_0-ones((2*NN+1)^2,1)*qb0;
K_2=K_0+ones((2*NN+1)^2,1)*qb0;
NNK1=find(abs(K_1(:,1)+i*K_1(:,2))<=0.8*NN*max(norm(b1m0),norm(b2m0))
);
NNK2=find(abs(K_2(:,1)+i*K_2(:,2))<=0.8*NN*max(norm(b1m0),norm(b2m0))
);

TKqb=t_vert/3*[1,1;1,1];
TKqtr=t_vert/3*[1,exp(-i*2*pi/3*(1+sqrt(3)*EExx*EExy+sqrt(3)*EEyy*EEx
y-EExy^2-EEyy^2));

exp(i*2*pi/3*(1+sqrt(3)*EExx*EExy+sqrt(3)*EEyy*EExy-EExy^2-EEyy^2)),1
];
TKqtl=t_vert/3*[1,exp(i*2*pi/3*(1-sqrt(3)*EExx*EExy-sqrt(3)*EEyy*EExy
-EExy^2-EEyy^2));

exp(-i*2*pi/3*(1-sqrt(3)*EExx*EExy-sqrt(3)*EEyy*EExy-EExy^2-EEyy^2)),
1];
```

```
T_Kqb=t_vert/3*[1,1;1,1];
T_Kqtr=t_vert/3*[1,exp(i*2*pi/3*(1+sqrt(3)*EExx*EExy+sqrt(3)*EEyy*EEx
y-EExy^2-EEyy^2));

exp(-i*2*pi/3*(1+sqrt(3)*EExx*EExy+sqrt(3)*EEyy*EExy-EExy^2-EEyy^2)),
1];
T_Kqtl=t_vert/3*[1,exp(-i*2*pi/3*(1-sqrt(3)*EExx*EExy-sqrt(3)*EEyy*EE
xy-EExy^2-EEyy^2));

exp(i*2*pi/3*(1-sqrt(3)*EExx*EExy-sqrt(3)*EEyy*EExy-EExy^2-EEyy^2)),1
];
qb=-4*pi/(3*sqrt(3)*d)*[EExx*cos(theta/2)+EExy*sin(theta/2),(2-EExx)*
sin(theta/2)+EExy*cos(theta/2)];
qtr=2*pi/(9*d)*[(sqrt(3)*EExx-3*EExy)*cos(theta/2)+(6-3*EEyy+sqrt(3)*
EExy)*sin(theta/2),-(3*EEyy-sqrt(3)*EExy)*cos(theta/2)+(2*sqrt(3)-sqr
t(3)*EExx+3*EExy)*sin(theta/2)];
qtl=2*pi/(9*d)*[(sqrt(3)*EExx+3*EExy)*cos(theta/2)-(6-3*EEyy-sqrt(3)*
EExy)*sin(theta/2),(3*EEyy+sqrt(3)*EExy)*cos(theta/2)+(2*sqrt(3)-sqrt
(3)*EExx-3*EExy)*sin(theta/2)];
b1m=2*pi/(3*d)*[-(sqrt(3)*EExx+EExy)*cos(theta/2)+(2-EEyy-sqrt(3)*EEx
y)*sin(theta/2),-(EEyy+sqrt(3)*EExy)*cos(theta/2)-(2*sqrt(3)-sqrt(3)*
EExx-EExy)*sin(theta/2)];
b2m=2*pi/(3*d)*[(sqrt(3)*EExx-EExy)*cos(theta/2)+(2-EEyy+sqrt(3)*EExy
)*sin(theta/2),-(EEyy-sqrt(3)*EExy)*cos(theta/2)+(2*sqrt(3)-sqrt(3)*E
Exx+EExy)*sin(theta/2)];
ZZ=zeros(2);

[NN1,NN2]=meshgrid(-NN:NN);
NN1=reshape(NN1,(2*NN+1)^2,1);
NN2=reshape(NN2,(2*NN+1)^2,1);
K0=NN1*b1m+NN2*b2m;
K1=K0-ones((2*NN+1)^2,1)*qb;
K2=K0+ones((2*NN+1)^2,1)*qb;
K1=K1(NNK1,:);
K2=K2(NNK2,:);
NK1=length(K1);
NK2=length(K2);

kx1=linspace(qtr(1),qtr(1)+qb(1),50);
ky1=linspace(qtr(2),qtr(2)+qb(2),50);
kx2=linspace(qtr(1)+qb(1),0,50);
ky2=linspace(qtr(2)+qb(2),0,50);
kx3=linspace(0,qtr(1)+1/2*qb(1),50);
ky3=linspace(0,qtr(2)+1/2*qb(2),50);
```

```
kx4=linspace(qtr(1)+1/2*qb(1),qtr(1),50);
ky4=linspace(qtr(2)+1/2*qb(2),qtr(2),50);
kx=[kx1,kx2,kx3,kx4];
ky=[ky1,ky2,ky3,ky4];

for n1=1:length(K1)
    for n2=1:length(K2)
        if norm((K1(n1,:)-K2(n2,:))+qb)<1/2*norm(qb)
            HK_T(2*n1-1:2*n1,2*n2-1:2*n2)=TKqb;
            H_K_T(2*n1-1:2*n1,2*n2-1:2*n2)=T_Kqb';
        elseif norm((K1(n1,:)-K2(n2,:))-qb)<1/2*norm(qb)
            HK_T(2*n1-1:2*n1,2*n2-1:2*n2)=TKqb';
            H_K_T(2*n1-1:2*n1,2*n2-1:2*n2)=T_Kqb;
        elseif norm((K1(n1,:)-K2(n2,:))+qtr)<1/2*norm(qtr)
            HK_T(2*n1-1:2*n1,2*n2-1:2*n2)=TKqtr;
            H_K_T(2*n1-1:2*n1,2*n2-1:2*n2)=T_Kqtr';
        elseif norm((K1(n1,:)-K2(n2,:))-qtr)<1/2*norm(qtr)
            HK_T(2*n1-1:2*n1,2*n2-1:2*n2)=TKqtr';
            H_K_T(2*n1-1:2*n1,2*n2-1:2*n2)=T_Kqtr;
        elseif norm((K1(n1,:)-K2(n2,:))+qtl)<1/2*norm(qtl)
            HK_T(2*n1-1:2*n1,2*n2-1:2*n2)=TKqtl;
            H_K_T(2*n1-1:2*n1,2*n2-1:2*n2)=T_Kqtl';
        elseif norm((K1(n1,:)-K2(n2,:))-qtl)<1/2*norm(qtl)
            HK_T(2*n1-1:2*n1,2*n2-1:2*n2)=TKqtl';
            H_K_T(2*n1-1:2*n1,2*n2-1:2*n2)=T_Kqtl;
        else
            HK_T(2*n1-1:2*n1,2*n2-1:2*n2)=ZZ;
            H_K_T(2*n1-1:2*n1,2*n2-1:2*n2)=ZZ;
        end
    end
end
for n=1:length(kx)
    q=[kx(n),ky(n)];
    for n1=1:length(K1)

qK1=((1+EExx)*(q(1)-K1(n1,1)+A(1))+EExy*(q(2)-K1(n1,2)+A(2)))+i*(EExy
*(q(1)-K1(n1,1)+A(1))+(1+EEyy)*(q(2)-K1(n1,2)+A(2)));
        HK1(2*n1-1:2*n1,2*n1-1:2*n1)=hbar_vf*[0,
conj(qK1)*exp(i*theta/2);
            qK1*exp(-i*theta/2), 0]+MM1*[1,0;0,-1];
        q_K2=q(1)-K1(n1,1)+i*(q(2)-K1(n1,2));
        H_K2(2*n1-1:2*n1,2*n1-1:2*n1)=-hbar_vf*[0, q_K2*exp(i*theta/2);
            conj(q_K2)*exp(-i*theta/2), 0]+MM2*[1,0;0,-1];
    end
```

```
    for n1=1:length(K2)
        qK2=q(1)-K2(n1,1)+i*(q(2)-K2(n1,2));
        HK2(2*n1-1:2*n1,2*n1-1:2*n1)=hbar_vf*[0,
conj(qK2)*exp(-i*theta/2);
            qK2*exp(i*theta/2), 0]+MM2*[1,0;0,-1];

q_K1=((1+EExx)*(q(1)-K2(n1,1)-A(1))+EExy*(q(2)-K2(n1,2)-A(2)))+i*(EEx
y*(q(1)-K2(n1,1)-A(1))+(1+EEyy)*(q(2)-K2(n1,2)-A(2)));
        H_K1(2*n1-1:2*n1,2*n1-1:2*n1)=-hbar_vf*[0,
q_K1*exp(-i*theta/2);
            conj(q_K1)*exp(i*theta/2), 0]+MM1*[1,0;0,-1];
    end
    HK=[HK1,HK_T;HK_T',HK2];
    H_K=[H_K2,H_K_T;H_K_T',H_K1];
    EK(:,n)=eig(HK);
    E_K(:,n)=eig(H_K);
    n
end
hold all
plot(linspace(0,4,length(kx)),EK(NK1+NK2-1,:)*1e3,'c--',linspace(0,4,
length(kx)),EK(NK1+NK2,:)*1e3,'b--',linspace(0,4,length(kx)),EK(NK1+N
K2+1,:)*1e3,'r--',linspace(0,4,length(kx)),EK(NK1+NK2+2,:)*1e3,'m--',
'LineWidth',2),colormap(jet)
```

Orbital magnetic moment in Brillouin zone

```
e=1;%unit: e
hbar=6.582*1e-16;%unit: eV*s
mu_b=5.788*1e-5*1e18;%unit: eV*nm^2/(V*s);

theta=1.2/180*pi;
t=2.8;
MM1=1*0.017;
MM2=-0;
d=1.42;
hbar_vf=3/2*d*t;
t_vert=0.33;
epsilon=1*1e-3;
phi=-0*theta/2+0*pi/5+0*pi/3;
nu=0.165;
Epsilon=epsilon*[cos(phi),-sin(phi);sin(phi),cos(phi)]*[-1,0;0,nu]*[c
os(phi),sin(phi);-sin(phi),cos(phi)];
EExx=Epsilon(1,1);
EEyy=Epsilon(2,2);
EExy=Epsilon(1,2);
beta=1.57;
A=beta/d*[EExx-EEyy,-2*EExy];

qb0=8*pi*sin(1/2*theta)/(3*sqrt(3)*d)*[0,-1];
qtr0=8*pi*sin(1/2*theta)/(3*sqrt(3)*d)*[sqrt(3)/2,1/2];
qtl0=8*pi*sin(1/2*theta)/(3*sqrt(3)*d)*[-sqrt(3)/2,1/2];
b1m0=8*pi*sin(1/2*theta)/(3*d)*[1/2,-sqrt(3)/2];
b2m0=8*pi*sin(1/2*theta)/(3*d)*[1/2,sqrt(3)/2];

NN=3;
[NN1,NN2]=meshgrid(-NN:NN);
NN1=reshape(NN1,(2*NN+1)^2,1);
NN2=reshape(NN2,(2*NN+1)^2,1);
K_0=NN1*b1m0+NN2*b2m0;
K_1=K_0-ones((2*NN+1)^2,1)*qb0;
K_2=K_0+ones((2*NN+1)^2,1)*qb0;
NNK1=find(abs(K_1(:,1)+i*K_1(:,2))<=0.8*NN*max(norm(b1m0),norm(b2m0))
```

```
);
NNK2=find(abs(K_2(:,1)+i*K_2(:,2))<=0.8*NN*max(norm(b1m0),norm(b2m0))
);

TKqb=t_vert/3*[1,1;1,1];
TKqtr=t_vert/3*[1,exp(-i*2*pi/3*(1+sqrt(3)*EExx*EExy+sqrt(3)*EEyy*EEx
y-EExy^2-EEyy^2));

exp(i*2*pi/3*(1+sqrt(3)*EExx*EExy+sqrt(3)*EEyy*EExy-EExy^2-EEyy^2)),1
];
TKqtl=t_vert/3*[1,exp(i*2*pi/3*(1-sqrt(3)*EExx*EExy-sqrt(3)*EEyy*EExy
-EExy^2-EEyy^2));

exp(-i*2*pi/3*(1-sqrt(3)*EExx*EExy-sqrt(3)*EEyy*EExy-EExy^2-EEyy^2)),
1];
T_Kqb=t_vert/3*[1,1;1,1];
T_Kqtr=t_vert/3*[1,exp(i*2*pi/3*(1+sqrt(3)*EExx*EExy+sqrt(3)*EEyy*EEx
y-EExy^2-EEyy^2));

exp(-i*2*pi/3*(1+sqrt(3)*EExx*EExy+sqrt(3)*EEyy*EExy-EExy^2-EEyy^2)),
1];
T_Kqtl=t_vert/3*[1,exp(-i*2*pi/3*(1-sqrt(3)*EExx*EExy-sqrt(3)*EEyy*EE
xy-EExy^2-EEyy^2));

exp(i*2*pi/3*(1-sqrt(3)*EExx*EExy-sqrt(3)*EEyy*EExy-EExy^2-EEyy^2)),1
];
qb=-4*pi/(3*sqrt(3)*d)*[EExx*cos(theta/2)+EExy*sin(theta/2),(2-EExx)*
sin(theta/2)+EExy*cos(theta/2)];
qtr=2*pi/(9*d)*[(sqrt(3)*EExx-3*EExy)*cos(theta/2)+(6-3*EEyy+sqrt(3)*
EExy)*sin(theta/2),-(3*EEyy-sqrt(3)*EExy)*cos(theta/2)+(2*sqrt(3)-sqr
t(3)*EExx+3*EExy)*sin(theta/2)];
qtl=2*pi/(9*d)*[(sqrt(3)*EExx+3*EExy)*cos(theta/2)-(6-3*EEyy-sqrt(3)*
EExy)*sin(theta/2),(3*EEyy+sqrt(3)*EExy)*cos(theta/2)+(2*sqrt(3)-sqrt
(3)*EExx-3*EExy)*sin(theta/2)];
b1m=2*pi/(3*d)*[-(sqrt(3)*EExx+EExy)*cos(theta/2)+(2-EEyy-sqrt(3)*EEx
y)*sin(theta/2),-(EEyy+sqrt(3)*EExy)*cos(theta/2)-(2*sqrt(3)-sqrt(3)*
EExx-EExy)*sin(theta/2)];
b2m=2*pi/(3*d)*[(sqrt(3)*EExx-EExy)*cos(theta/2)+(2-EEyy+sqrt(3)*EExy
)*sin(theta/2),-(EEyy-sqrt(3)*EExy)*cos(theta/2)+(2*sqrt(3)-sqrt(3)*E
Exx+EExy)*sin(theta/2)];
ZZ=zeros(2);

[NN1,NN2]=meshgrid(-NN:NN);
NN1=reshape(NN1,(2*NN+1)^2,1);
```

```
NN2=reshape(NN2,(2*NN+1)^2,1);
K0=NN1*b1m+NN2*b2m;
K1=K0-ones((2*NN+1)^2,1)*qb;
K2=K0+ones((2*NN+1)^2,1)*qb;
K1=K1(NNK1,:);
K2=K2(NNK2,:);
NK1=length(K1);
NK2=length(K2);
%figure
%plot(K1(:,1)',K1(:,2)','r*',K2(:,1)',K2(:,2)','bo'),axis equal

KX_max=max([qb(1),qb(1)+qtr(1),qtr(1),-qb(1),qtl(1),qtl(1)+qb(1)]);
KX_min=min([qb(1),qb(1)+qtr(1),qtr(1),-qb(1),qtl(1),qtl(1)+qb(1)]);
KY_max=max([qb(2),qb(2)+qtr(2),qtr(2),-qb(2),qtl(2),qtl(2)+qb(2)]);
KY_min=min([qb(2),qb(2)+qtr(2),qtr(2),-qb(2),qtl(2),qtl(2)+qb(2)]);
Num=201;
[kx,ky]=meshgrid(1.2*linspace(KX_min,KX_max,Num),1.2*linspace(KY_min,
KY_max,Num));

for n1=1:length(K1)
    for n2=1:length(K2)
        if norm((K1(n1,:)-K2(n2,:))+qb)<1/2*norm(qb)
            HK_T(2*n1-1:2*n1,2*n2-1:2*n2)=TKqb;
            H_K_T(2*n1-1:2*n1,2*n2-1:2*n2)=T_Kqb';
        elseif norm((K1(n1,:)-K2(n2,:))-qb)<1/2*norm(qb)
            HK_T(2*n1-1:2*n1,2*n2-1:2*n2)=TKqb';
            H_K_T(2*n1-1:2*n1,2*n2-1:2*n2)=T_Kqb;
        elseif norm((K1(n1,:)-K2(n2,:))+qtr)<1/2*norm(qtr)
            HK_T(2*n1-1:2*n1,2*n2-1:2*n2)=TKqtr;
            H_K_T(2*n1-1:2*n1,2*n2-1:2*n2)=T_Kqtr';
        elseif norm((K1(n1,:)-K2(n2,:))-qtr)<1/2*norm(qtr)
            HK_T(2*n1-1:2*n1,2*n2-1:2*n2)=TKqtr';
            H_K_T(2*n1-1:2*n1,2*n2-1:2*n2)=T_Kqtr;
        elseif norm((K1(n1,:)-K2(n2,:))+qtl)<1/2*norm(qtl)
            HK_T(2*n1-1:2*n1,2*n2-1:2*n2)=TKqtl;
            H_K_T(2*n1-1:2*n1,2*n2-1:2*n2)=T_Kqtl';
        elseif norm((K1(n1,:)-K2(n2,:))-qtl)<1/2*norm(qtl)
            HK_T(2*n1-1:2*n1,2*n2-1:2*n2)=TKqtl';
            H_K_T(2*n1-1:2*n1,2*n2-1:2*n2)=T_Kqtl;
        else
            HK_T(2*n1-1:2*n1,2*n2-1:2*n2)=ZZ;
            H_K_T(2*n1-1:2*n1,2*n2-1:2*n2)=ZZ;
        end
    end
```

```
end

for nn=1:length(kx)
    for mm=1:length(ky)
        q=[kx(nn,mm),ky(nn,mm)];
        for n1=1:length(K1)

qK1=((1+EExx)*(q(1)-K1(n1,1)+A(1))+EExy*(q(2)-K1(n1,2)+A(2)))+i*(EExy
*(q(1)-K1(n1,1)+A(1))+(1+EEyy)*(q(2)-K1(n1,2)+A(2)));
            HK1(2*n1-1:2*n1,2*n1-1:2*n1)=hbar_vf*[0,
conj(qK1)*exp(i*theta/2);
                qK1*exp(-i*theta/2), 0]+MM1*[1,0;0,-1];
            q_K2=q(1)-K1(n1,1)+i*(q(2)-K1(n1,2));
            H_K2(2*n1-1:2*n1,2*n1-1:2*n1)=-hbar_vf*[0,
q_K2*exp(i*theta/2);
                conj(q_K2)*exp(-i*theta/2), 0]+MM2*[1,0;0,-1];
        end
        for n1=1:length(K2)
            qK2=q(1)-K2(n1,1)+i*(q(2)-K2(n1,2));
            HK2(2*n1-1:2*n1,2*n1-1:2*n1)=hbar_vf*[0,
conj(qK2)*exp(-i*theta/2);
                qK2*exp(i*theta/2), 0]+MM2*[1,0;0,-1];

q_K1=((1+EExx)*(q(1)-K2(n1,1)-A(1))+EExy*(q(2)-K2(n1,2)-A(2)))+i*(EEx
y*(q(1)-K2(n1,1)-A(1))+(1+EEyy)*(q(2)-K2(n1,2)-A(2)));
            H_K1(2*n1-1:2*n1,2*n1-1:2*n1)=-hbar_vf*[0,
q_K1*exp(-i*theta/2);
                conj(q_K1)*exp(i*theta/2), 0]+MM1*[1,0;0,-1];
        end
        HK=[HK1,HK_T;HK_T',HK2];
        H_K=[H_K2,H_K_T;H_K_T',H_K1];
        [uK,vK]=eig(HK);
        [AK,BK]=sort(diag(vK),'ascend');
        [u_K,v_K]=eig(H_K);
        [A_K,B_K]=sort(diag(v_K),'ascend');
        E1(nn,mm)=AK(NK1+NK2);
        E2(nn,mm)=AK(NK1+NK2+1);
        E_1(nn,mm)=A_K(NK1+NK2);
        E_2(nn,mm)=A_K(NK1+NK2+1);
    end
end

Ec=[-2.25,-2.15,-2.05,-1.95,-1.75,-1.55,-1.3,-1,0,1,2,3,4,5,7,9]*1e-3
;
```

```
figure
CK=contour(kx,ky,E2,Ec,'ShowText','on');
CK(:,find(CK(2,:)>=1))=[nan;nan]*ones(1,length(find(CK(2,:)>=1)));
length(CK)

for nn2=1:length(CK)
    if isnan(CK(1,nn2))
        MK_z(nn2)=nan;
    else
        q=[CK(1,nn2),CK(2,nn2)];
        for n1=1:length(K1)

qK1=((1+EExx)*(q(1)-K1(n1,1)+A(1))+EExy*(q(2)-K1(n1,2)+A(2)))+i*(EExy
*(q(1)-K1(n1,1)+A(1))+(1+EEyy)*(q(2)-K1(n1,2)+A(2)));
            HK1(2*n1-1:2*n1,2*n1-1:2*n1)=hbar_vf*[0,
conj(qK1)*exp(i*theta/2);
                qK1*exp(-i*theta/2), 0]+MM1*[1,0;0,-1];
            HK1_diff_x(2*n1-1:2*n1,2*n1-1:2*n1)=hbar_vf*[0,
((1+EExx)-i*EExy)*exp(i*theta/2);
                ((1+EExx)+i*EExy)*exp(-i*theta/2), 0];
            HK1_diff_y(2*n1-1:2*n1,2*n1-1:2*n1)=hbar_vf*[0,
(EExy-i*(1+EEyy))*exp(i*theta/2);
                (EExy+i*(1+EEyy))*exp(-i*theta/2), 0];
        end
        for n1=1:length(K2)
            qK2=q(1)-K2(n1,1)+i*(q(2)-K2(n1,2));
            HK2(2*n1-1:2*n1,2*n1-1:2*n1)=hbar_vf*[0,
conj(qK2)*exp(-i*theta/2);
                qK2*exp(i*theta/2), 0]+MM2*[1,0;0,-1];
            HK2_diff_x(2*n1-1:2*n1,2*n1-1:2*n1)=hbar_vf*[0,
exp(-i*theta/2);
                exp(i*theta/2), 0];
            HK2_diff_y(2*n1-1:2*n1,2*n1-1:2*n1)=hbar_vf*[0,
-i*exp(-i*theta/2);
                i*exp(i*theta/2), 0];
        end
        HK=[HK1,HK_T;HK_T',HK2];

HK_diff_x=[HK1_diff_x,zeros(2*NK1,2*NK2);zeros(2*NK1,2*NK2),HK2_diff_
x];

HK_diff_y=[HK1_diff_y,zeros(2*NK1,2*NK2);zeros(2*NK1,2*NK2),HK2_diff_
y];
        [uK,vK]=eig(HK);
```

```
        [AK,BK]=sort(diag(vK),'ascend');


WW=-1./(repmat(AK,[1,2*(NK1+NK2)])-repmat(AK,[1,2*(NK1+NK2)])');
        WW(isinf(WW))=0;

OrbitalK_mz=-1/2*imag((uK(:,BK(NK1+NK2+1))'*HK_diff_x*uK(:,BK).*WW(NK
1+NK2+1,:)).*transpose(uK(:,BK)'*HK_diff_y*uK(:,BK(NK1+NK2+1)))...

-(uK(:,BK(NK1+NK2+1))'*HK_diff_y*uK(:,BK).*WW(NK1+NK2+1,:)).*transpos
e(uK(:,BK)'*HK_diff_x*uK(:,BK(NK1+NK2+1))));
        MK_z(nn2)=e/hbar*0.1^2*sum(OrbitalK_mz,2)/mu_b;

    end
end

E_c=[-2.25,-2.15,-2.05,-1.95,-1.75,-1.55,-1.3,-1,0,1,2,3,4,5,7,9]*1e-
3;
figure
C_K=contour(kx,ky,E_2,E_c,'ShowText','on');
C_K(:,find(C_K(2,:)>=1))=[nan;nan]*ones(1,length(find(C_K(2,:)>=1)));
length(C_K)

for nn3=1:length(C_K)
    if isnan(C_K(1,nn3))
        M_K_z(nn3)=nan;
    else
        q=[C_K(1,nn3),C_K(2,nn3)];
        for n1=1:length(K1)
            q_K2=q(1)-K1(n1,1)+i*(q(2)-K1(n1,2));
            H_K2(2*n1-1:2*n1,2*n1-1:2*n1)=-hbar_vf*[0,
q_K2*exp(i*theta/2);
                conj(q_K2)*exp(-i*theta/2), 0]+MM2*[1,0;0,-1];
            H_K2_diff_x(2*n1-1:2*n1,2*n1-1:2*n1)=-hbar_vf*[0,
exp(i*theta/2);
                exp(-i*theta/2), 0];
            H_K2_diff_y(2*n1-1:2*n1,2*n1-1:2*n1)=-hbar_vf*[0,
i*exp(i*theta/2);
                -i*exp(-i*theta/2), 0];
        end
        for n1=1:length(K2)

q_K1=((1+EExx)*(q(1)-K2(n1,1)-A(1))+EExy*(q(2)-K2(n1,2)-A(2)))+i*(EEx
y*(q(2)-K2(n1,2)-A(1))+(1+EEyy)*(q(2)-K2(n1,2)-A(2)));
```

```
            H_K1(2*n1-1:2*n1,2*n1-1:2*n1)=-hbar_vf*[0,
q_K1*exp(-i*theta/2);
                conj(q_K1)*exp(i*theta/2), 0]+MM1*[1,0;0,-1];
            H_K1_diff_x(2*n1-1:2*n1,2*n1-1:2*n1)=-hbar_vf*[0,
((1+EExx)+i*EExy)*exp(-i*theta/2);
                ((1+EExx)-i*EExy)*exp(i*theta/2), 0];
            H_K1_diff_y(2*n1-1:2*n1,2*n1-1:2*n1)=-hbar_vf*[0,
(EExy+i*(1+EEyy))*exp(-i*theta/2);
                (EExy-i*(1+EEyy))*exp(i*theta/2), 0];
        end
        H_K=[H_K2,H_K_T;H_K_T',H_K1];

H_K_diff_x=[H_K2_diff_x,zeros(2*NK1,2*NK2);zeros(2*NK1,2*NK2),H_K1_di
ff_x];

H_K_diff_y=[H_K2_diff_y,zeros(2*NK1,2*NK2);zeros(2*NK1,2*NK2),H_K1_di
ff_y];
        [u_K,v_K]=eig(H_K);
        [A_K,B_K]=sort(diag(v_K),'ascend');


WW=-1./(repmat(A_K,[1,2*(NK1+NK2)])-repmat(A_K,[1,2*(NK1+NK2)])');
        WW(isinf(WW))=0;

Orbital_K_mz=-1/2*imag((u_K(:,B_K(NK1+NK2+1))'*H_K_diff_x*u_K(:,B_K).
*WW(NK1+NK2+1,:)).*transpose(u_K(:,B_K)'*H_K_diff_y*u_K(:,B_K(NK1+NK2
+1)))...

-(u_K(:,B_K(NK1+NK2+1))'*H_K_diff_y*u_K(:,B_K).*WW(NK1+NK2+1,:)).*tra
nspose(u_K(:,B_K)'*H_K_diff_x*u_K(:,B_K(NK1+NK2+1))));

        M_K_z(nn3)=e/hbar*0.1^2*sum(Orbital_K_mz,2)/mu_b;

    end
end

Max_Mz=max([MK_z,M_K_z]);
Min_Mz=min([MK_z,M_K_z]);

figure
plot([qb(1),qb(1)+qtr(1),qtr(1),-qb(1),qtl(1),qtl(1)+qb(1),qb(1)],[qb
(2),qb(2)+qtr(2),qtr(2),-qb(2),qtl(2),qtl(2)+qb(2),qb(2)],'k-','LineW
idth',2),axis equal
hold on
```

```
patch([CK(1,:),nan],[CK(2,:),nan],[MK_z,nan],'edgecolor','flat','face
color','none','LineWidth',2)
axis equal
colormap(jet)
c=colorbar;
c.Position(4)=0.72;
caxis([Min_Mz,Max_Mz])
text=annotation('textbox');
text.String='m_z (\mu_b)';
text.FontName='Times New Roman';
text.FontSize=21;
text.FontWeight='b';
text.EdgeColor='none';
caxis([-30,30])
c.Ticks=[-30,-20,-10,0,10,20,30];
axis off
set(gca,'FontName','Times New Roman','FontSize', 18,'fontweight','b')
```

Magnetoelectric susceptibility

```
e=1;%unit: e
hbar=6.582*1e-16;%unit: eV*s
mu_b=5.788*1e-5*1e18;%unit: eV*nm^2/(V*s);
kb=8.617*1e-5;%unit: eV/K
T=5;%unit: K
beta0=1/(kb*T);
mu_0=1.256*1e-6;%unit: T*m/A
tau=10*1e-12;%unit: s

theta=1.2/180*pi;
t=2.8;
MM1=1*0.017;
MM2=-0;
d=1.42;%Angstrom
hbar_vf=3/2*d*t;
t_vert=0.33;
epsilon=1*1e-3;
phi=-0*theta/2+0*pi/5+0*pi/3;
nu=0.165;
Epsilon=epsilon*[cos(phi),-sin(phi);sin(phi),cos(phi)]*[-1,0;0,nu]*[c
os(phi),sin(phi);-sin(phi),cos(phi)];
EExx=Epsilon(1,1);
EEyy=Epsilon(2,2);
EExy=Epsilon(1,2);
beta=1.57;
A=beta/d*[EExx-EEyy,-2*EExy];

qb0=8*pi*sin(1/2*theta)/(3*sqrt(3)*d)*[0,-1];
qtr0=8*pi*sin(1/2*theta)/(3*sqrt(3)*d)*[sqrt(3)/2,1/2];
qtl0=8*pi*sin(1/2*theta)/(3*sqrt(3)*d)*[-sqrt(3)/2,1/2];
b1m0=8*pi*sin(1/2*theta)/(3*d)*[1/2,-sqrt(3)/2];
b2m0=8*pi*sin(1/2*theta)/(3*d)*[1/2,sqrt(3)/2];

NN=3;
[NN1,NN2]=meshgrid(-NN:NN);
NN1=reshape(NN1,(2*NN+1)^2,1);
NN2=reshape(NN2,(2*NN+1)^2,1);
K_0=NN1*b1m0+NN2*b2m0;
```

```
K_1=K_0-ones((2*NN+1)^2,1)*qb0;
K_2=K_0+ones((2*NN+1)^2,1)*qb0;
NNK1=find(abs(K_1(:,1)+i*K_1(:,2))<=0.8*NN*max(norm(b1m0),norm(b2m0))
);
NNK2=find(abs(K_2(:,1)+i*K_2(:,2))<=0.8*NN*max(norm(b1m0),norm(b2m0))
);

TKqb=t_vert/3*[1,1;1,1];
TKqtr=t_vert/3*[1,exp(-i*2*pi/3*(1+sqrt(3)*EExx*EExy+sqrt(3)*EEyy*EEx
y-EExy^2-EEyy^2));

exp(i*2*pi/3*(1+sqrt(3)*EExx*EExy+sqrt(3)*EEyy*EExy-EExy^2-EEyy^2)),1
];
TKqtl=t_vert/3*[1,exp(i*2*pi/3*(1-sqrt(3)*EExx*EExy-sqrt(3)*EEyy*EExy
-EExy^2-EEyy^2));

exp(-i*2*pi/3*(1-sqrt(3)*EExx*EExy-sqrt(3)*EEyy*EExy-EExy^2-EEyy^2)),
1];
T_Kqb=t_vert/3*[1,1;1,1];
T_Kqtr=t_vert/3*[1,exp(i*2*pi/3*(1+sqrt(3)*EExx*EExy+sqrt(3)*EEyy*EEx
y-EExy^2-EEyy^2));

exp(-i*2*pi/3*(1+sqrt(3)*EExx*EExy+sqrt(3)*EEyy*EExy-EExy^2-EEyy^2)),
1];
T_Kqtl=t_vert/3*[1,exp(-i*2*pi/3*(1-sqrt(3)*EExx*EExy-sqrt(3)*EEyy*EE
xy-EExy^2-EEyy^2));

exp(i*2*pi/3*(1-sqrt(3)*EExx*EExy-sqrt(3)*EEyy*EExy-EExy^2-EEyy^2)),1
];
qb=-4*pi/(3*sqrt(3)*d)*[EExx*cos(theta/2)+EExy*sin(theta/2),(2-EExx)*
sin(theta/2)+EExy*cos(theta/2)];
qtr=2*pi/(9*d)*[(sqrt(3)*EExx-3*EExy)*cos(theta/2)+(6-3*EEyy+sqrt(3)*
EExy)*sin(theta/2),-(3*EEyy-sqrt(3)*EExy)*cos(theta/2)+(2*sqrt(3)-sqr
t(3)*EExx+3*EExy)*sin(theta/2)];
qtl=2*pi/(9*d)*[(sqrt(3)*EExx+3*EExy)*cos(theta/2)-(6-3*EEyy-sqrt(3)*
EExy)*sin(theta/2),(3*EEyy+sqrt(3)*EExy)*cos(theta/2)+(2*sqrt(3)-sqrt
(3)*EExx-3*EExy)*sin(theta/2)];
b1m=2*pi/(3*d)*[-(sqrt(3)*EExx+EExy)*cos(theta/2)+(2-EEyy-sqrt(3)*EEx
y)*sin(theta/2),-(EEyy+sqrt(3)*EExy)*cos(theta/2)-(2*sqrt(3)-sqrt(3)*
EExx-EExy)*sin(theta/2)];
b2m=2*pi/(3*d)*[(sqrt(3)*EExx-EExy)*cos(theta/2)+(2-EEyy+sqrt(3)*EExy
)*sin(theta/2),-(EEyy-sqrt(3)*EExy)*cos(theta/2)+(2*sqrt(3)-sqrt(3)*E
Exx+EExy)*sin(theta/2)];
V_BZ=norm(cross([b1m,0],[b2m,0]));
```

```
ZZ=zeros(2);

[NN1,NN2]=meshgrid(-NN:NN);
NN1=reshape(NN1,(2*NN+1)^2,1);
NN2=reshape(NN2,(2*NN+1)^2,1);
K0=NN1*b1m+NN2*b2m;
K1=K0-ones((2*NN+1)^2,1)*qb;
K2=K0+ones((2*NN+1)^2,1)*qb;
K1=K1(NNK1,:);
K2=K2(NNK2,:);
NK1=length(K1);
NK2=length(K2);

KX_max=max([qb(1),qb(1)+qtr(1),qtr(1),-qb(1),qtl(1),qtl(1)+qb(1)]);
KX_min=min([qb(1),qb(1)+qtr(1),qtr(1),-qb(1),qtl(1),qtl(1)+qb(1)]);
KY_max=max([qb(2),qb(2)+qtr(2),qtr(2),-qb(2),qtl(2),qtl(2)+qb(2)]);
KY_min=min([qb(2),qb(2)+qtr(2),qtr(2),-qb(2),qtl(2),qtl(2)+qb(2)]);
Num=201;
[kx,ky]=meshgrid(1.2*linspace(KX_min,KX_max,Num),1.2*linspace(KY_min,
KY_max,Num));

for n1=1:length(K1)
    for n2=1:length(K2)
        if norm((K1(n1,:)-K2(n2,:))+qb)<1/2*norm(qb)
            HK_T(2*n1-1:2*n1,2*n2-1:2*n2)=TKqb;
            H_K_T(2*n1-1:2*n1,2*n2-1:2*n2)=T_Kqb';
        elseif norm((K1(n1,:)-K2(n2,:))-qb)<1/2*norm(qb)
            HK_T(2*n1-1:2*n1,2*n2-1:2*n2)=TKqb';
            H_K_T(2*n1-1:2*n1,2*n2-1:2*n2)=T_Kqb;
        elseif norm((K1(n1,:)-K2(n2,:))+qtr)<1/2*norm(qtr)
            HK_T(2*n1-1:2*n1,2*n2-1:2*n2)=TKqtr;
            H_K_T(2*n1-1:2*n1,2*n2-1:2*n2)=T_Kqtr';
        elseif norm((K1(n1,:)-K2(n2,:))-qtr)<1/2*norm(qtr)
            HK_T(2*n1-1:2*n1,2*n2-1:2*n2)=TKqtr';
            H_K_T(2*n1-1:2*n1,2*n2-1:2*n2)=T_Kqtr;
        elseif norm((K1(n1,:)-K2(n2,:))+qtl)<1/2*norm(qtl)
            HK_T(2*n1-1:2*n1,2*n2-1:2*n2)=TKqtl;
            H_K_T(2*n1-1:2*n1,2*n2-1:2*n2)=T_Kqtl';
        elseif norm((K1(n1,:)-K2(n2,:))-qtl)<1/2*norm(qtl)
            HK_T(2*n1-1:2*n1,2*n2-1:2*n2)=TKqtl';
            H_K_T(2*n1-1:2*n1,2*n2-1:2*n2)=T_Kqtl;
        else
            HK_T(2*n1-1:2*n1,2*n2-1:2*n2)=ZZ;
            H_K_T(2*n1-1:2*n1,2*n2-1:2*n2)=ZZ;
```

```
        end

        HK1_diff_x(2*n1-1:2*n1,2*n1-1:2*n1)=hbar_vf*[0,
((1+EExx)-i*EExy)*exp(i*theta/2);
            ((1+EExx)+i*EExy)*exp(-i*theta/2), 0];
        HK1_diff_y(2*n1-1:2*n1,2*n1-1:2*n1)=hbar_vf*[0,
(EExy-i*(1+EEyy))*exp(i*theta/2);
            (EExy+i*(1+EEyy))*exp(-i*theta/2), 0];

        H_K2_diff_x(2*n1-1:2*n1,2*n1-1:2*n1)=-hbar_vf*[0,
exp(i*theta/2);
            exp(-i*theta/2), 0];
        H_K2_diff_y(2*n1-1:2*n1,2*n1-1:2*n1)=-hbar_vf*[0,
i*exp(i*theta/2);
            -i*exp(-i*theta/2), 0];

        HK2_diff_x(2*n1-1:2*n1,2*n1-1:2*n1)=hbar_vf*[0,
exp(-i*theta/2);
            exp(i*theta/2), 0];
        HK2_diff_y(2*n1-1:2*n1,2*n1-1:2*n1)=hbar_vf*[0,
-i*exp(-i*theta/2);
            i*exp(i*theta/2), 0];

        H_K1_diff_x(2*n1-1:2*n1,2*n1-1:2*n1)=-hbar_vf*[0,
((1+EExx)+i*EExy)*exp(-i*theta/2);
            ((1+EExx)-i*EExy)*exp(i*theta/2), 0];
        H_K1_diff_y(2*n1-1:2*n1,2*n1-1:2*n1)=-hbar_vf*[0,
(EExy+i*(1+EEyy))*exp(-i*theta/2);
            (EExy-i*(1+EEyy))*exp(i*theta/2), 0];

    end
end
HK_diff_x=[HK1_diff_x,zeros(2*NK1,2*NK2);zeros(2*NK1,2*NK2),HK2_diff_
x];
HK_diff_y=[HK1_diff_y,zeros(2*NK1,2*NK2);zeros(2*NK1,2*NK2),HK2_diff_
y];
H_K_diff_x=[H_K2_diff_x,zeros(2*NK1,2*NK2);zeros(2*NK1,2*NK2),H_K1_di
ff_x];
H_K_diff_y=[H_K2_diff_y,zeros(2*NK1,2*NK2);zeros(2*NK1,2*NK2),H_K1_di
ff_y];

kkx=reshape(kx,Num^2,1);
kky=reshape(ky,Num^2,1);
kxv=[qb(1),qb(1)+qtr(1),qtr(1),-qb(1),qtl(1),qtl(1)+qb(1),qb(1)]';
```

```
kyv=[qb(2),qb(2)+qtr(2),qtr(2),-qb(2),qtl(2),qtl(2)+qb(2),qb(2)]';
[in,on]=inpolygon(kkx,kky,kxv,kyv);
kx_BZ=[kkx(in);kkx(on)];
ky_BZ=[kky(in);kky(on)];

for nn=1:length(kx_BZ)
    q=[kx_BZ(nn),ky_BZ(nn)];
    for n1=1:length(K1)

qK1=((1+EExx)*(q(1)-K1(n1,1)+A(1))+EExy*(q(2)-K1(n1,2)+A(2)))+i*(EExy
*(q(1)-K1(n1,1)+A(1))+(1+EEyy)*(q(2)-K1(n1,2)+A(2)));
        HK1(2*n1-1:2*n1,2*n1-1:2*n1)=hbar_vf*[0,
conj(qK1)*exp(i*theta/2);
            qK1*exp(-i*theta/2), 0]+MM1*[1,0;0,-1];
        q_K2=q(1)-K1(n1,1)+i*(q(2)-K1(n1,2));
        H_K2(2*n1-1:2*n1,2*n1-1:2*n1)=-hbar_vf*[0, q_K2*exp(i*theta/2);
            conj(q_K2)*exp(-i*theta/2), 0]+MM2*[1,0;0,-1];
    end
    for n1=1:length(K2)
        qK2=q(1)-K2(n1,1)+i*(q(2)-K2(n1,2));
        HK2(2*n1-1:2*n1,2*n1-1:2*n1)=hbar_vf*[0,
conj(qK2)*exp(-i*theta/2);
            qK2*exp(i*theta/2), 0]+MM2*[1,0;0,-1];

q_K1=((1+EExx)*(q(1)-K2(n1,1)-A(1))+EExy*(q(2)-K2(n1,2)-A(2)))+i*(EEx
y*(q(1)-K2(n1,1)-A(1))+(1+EEyy)*(q(2)-K2(n1,2)-A(2)));
        H_K1(2*n1-1:2*n1,2*n1-1:2*n1)=-hbar_vf*[0,
q_K1*exp(-i*theta/2);
            conj(q_K1)*exp(i*theta/2), 0]+MM1*[1,0;0,-1];
    end
    HK=[HK1,HK_T;HK_T',HK2];
    H_K=[H_K2,H_K_T;H_K_T',H_K1];
    [uK,vK]=eig(HK);
    [AK,BK]=sort(diag(vK),'ascend');
    EK(:,nn)=AK(NK1+NK2+1);
    [u_K,v_K]=eig(H_K);
    [A_K,B_K]=sort(diag(v_K),'ascend');
    E_K(:,nn)=A_K(NK1+NK2+1);

    WW=-1./(repmat(AK,[1,2*(NK1+NK2)])-repmat(AK,[1,2*(NK1+NK2)])');
    WW(isinf(WW))=0;

OrbitalK_mz=-1/2*imag((uK(:,BK(NK1+NK2+1))'*HK_diff_x*uK(:,BK).*WW(NK
1+NK2+1,:)).*transpose(uK(:,BK)'*HK_diff_y*uK(:,BK(NK1+NK2+1)))...
```

```
-(uK(:,BK(NK1+NK2+1))'*HK_diff_y*uK(:,BK).*WW(NK1+NK2+1,:)).*transpos
e(uK(:,BK)'*HK_diff_x*uK(:,BK(NK1+NK2+1))));


WW=-1./(repmat(A_K,[1,2*(NK1+NK2)])-repmat(A_K,[1,2*(NK1+NK2)])');
    WW(isinf(WW))=0;

Orbital_K_mz=-1/2*imag((u_K(:,B_K(NK1+NK2+1))'*H_K_diff_x*u_K(:,B_K).
*WW(NK1+NK2+1,:)).*transpose(u_K(:,B_K)'*H_K_diff_y*u_K(:,B_K(NK1+NK2
+1)))...

-(u_K(:,B_K(NK1+NK2+1))'*H_K_diff_y*u_K(:,B_K).*WW(NK1+NK2+1,:)).*tra
nspose(u_K(:,B_K)'*H_K_diff_x*u_K(:,B_K(NK1+NK2+1))));

    MK_z(:,nn)=e/hbar*0.1^2*sum(OrbitalK_mz,2)/mu_b;
    M_K_z(:,nn)=e/hbar*0.1^2*sum(Orbital_K_mz,2)/mu_b;

vKx(:,nn)=diag(uK(:,BK(NK1+NK2+1))'*HK_diff_x*uK(:,BK(NK1+NK2+1)));

vKy(:,nn)=diag(uK(:,BK(NK1+NK2+1))'*HK_diff_y*uK(:,BK(NK1+NK2+1)));

v_Kx(:,nn)=diag(u_K(:,BK(NK1+NK2+1))'*H_K_diff_x*u_K(:,BK(NK1+NK2+1))
);

v_Ky(:,nn)=diag(u_K(:,BK(NK1+NK2+1))'*H_K_diff_y*u_K(:,BK(NK1+NK2+1))
);
    %nn
end
Ef0=linspace(1.18*min(min([EK;E_K])),1.18*max(max([EK;E_K])),900);
for mm=1:length(Ef0)
    Ef=Ef0(mm);
    dEK=1/4*beta0*(1-tanh(1/2*beta0*(EK-Ef)).^2);
    dE_K=1/4*beta0*(1-tanh(1/2*beta0*(E_K-Ef)).^2);

aa_zx(mm)=-1/(4*pi^2)*e/hbar*sum(sum(MK_z.*vKx.*dEK+M_K_z.*v_Kx.*dE_K
))*V_BZ/length(kx_BZ)*tau*10;%unit:mu_b/(V*nm)

aa_zy(mm)=-1/(4*pi^2)*e/hbar*sum(sum(MK_z.*vKy.*dEK+M_K_z.*v_Ky.*dE_K
))*V_BZ/length(kx_BZ)*tau*10;%unit:mu_b/(V*nm);

Dos(mm)=1/(4*pi^2)*sum(sum(dEK+dE_K))*V_BZ/length(kx_BZ)*100;%unit:eV
^(-1)nm^(-2);
end
```

```
figure
yyaxis right
plot(Ef0*1e3,Dos,'r-','LineWidth',2)
set(gca,'YTick',[0,1,2,3],'YColor','r')
ylabel('DOS (eV^{-1}nm^{-2})','FontName','Times New
Roman','FontSize',36,'fontweight','b','Color','r')
set(gca,'LineWidth',1,'FontSize',28,'Fontname','Times New
Roman','fontweight','b')
yyaxis left
plot(Ef0*1e3,aa_zx,'b--',Ef0*1e3,aa_zy,'b-','LineWidth',2)
set(gca,'XTick',[-5,0,5,10,15])
set(gca,'YTick',[-1000,0,1000,2000],'YColor','b')
xlabel('E (meV)','FontName','Times New
Roman','FontSize',36,'fontweight','b');
ylabel('\alpha (\mu_bV^{-1}nm^{-1})','FontName','Times New
Roman','FontSize',36,'fontweight','b','Color','b')
set(gca,'LineWidth',1,'FontSize',28,'Fontname','Times New
Roman','fontweight','b')
axis([-5,16,-1500,2200])
legend({'\alpha_{zx}','\alpha_{zy}','DOS'},'FontSize',28,'FontName','
Times New Roman','fontweight','b')
legend('boxoff')

phi_E=linspace(0,2*pi,length(Ef0));
Ex=cos(phi_E);
Ey=sin(phi_E);
Mz=(Ex'*aa_zx+Ey'*aa_zy)*1e-5;%unit: mu_b/nm^2
[Err,E_phi]=meshgrid(linspace(0,1,length(Ef0)),linspace(0,2*pi,length
(Ef0)));
figure
pcolor(Err.*cos(E_phi),Err.*sin(E_phi),real(Mz)),shading interp
axis equal
axis off
text=annotation('textbox');
text.String='M_z (\mu_b/nm^2)';
text.FontName='Times New Roman';
text.FontSize=21;
text.FontWeight='b';
text.EdgeColor='none';
colormap(jet)
c=colorbar;
c.Ticks=[-0.02,-0.01,0,0.01,0.02];
c.Position(4)=0.72;
set(gca,'FontName','Times New Roman','FontSize', 18,'fontweight','b')
```

Coercive electric field

```
e=1;%unit: e
hbar=6.582*1e-16;%unit: eV*s
mu_b=5.788*1e-5*1e18;%unit: eV*nm^2/(V*s);
kb=8.617*1e-5;%unit: eV/K
T=5;%unit: K
beta0=1/(kb*T);
mu_0=1.256*1e-6;%unit: T*m/A
tau=10*1e-12;%unit: s

theta=1.2/180*pi;
t=2.8;
MM1=1*0.017;
MM2=-0;
d=1.42;%Angstrom
hbar_vf=3/2*d*t;
t_vert=0.33;
epsilon=1*1e-3;
phi=-0*theta/2+0*pi/5+0*pi/3;
nu=0.165;
Epsilon=epsilon*[cos(phi),-sin(phi);sin(phi),cos(phi)]*[-1,0;0,nu]*[c
os(phi),sin(phi);-sin(phi),cos(phi)];
EExx=Epsilon(1,1);
EEyy=Epsilon(2,2);
EExy=Epsilon(1,2);
beta=1.57;
A=beta/d*[EExx-EEyy,-2*EExy];

qb0=8*pi*sin(1/2*theta)/(3*sqrt(3)*d)*[0,-1];
```

```
qtr0=8*pi*sin(1/2*theta)/(3*sqrt(3)*d)*[sqrt(3)/2,1/2];
qtl0=8*pi*sin(1/2*theta)/(3*sqrt(3)*d)*[-sqrt(3)/2,1/2];
b1m0=8*pi*sin(1/2*theta)/(3*d)*[1/2,-sqrt(3)/2];
b2m0=8*pi*sin(1/2*theta)/(3*d)*[1/2,sqrt(3)/2];

NN=3;
[NN1,NN2]=meshgrid(-NN:NN);
NN1=reshape(NN1,(2*NN+1)^2,1);
NN2=reshape(NN2,(2*NN+1)^2,1);
K_0=NN1*b1m0+NN2*b2m0;
K_1=K_0-ones((2*NN+1)^2,1)*qb0;
K_2=K_0+ones((2*NN+1)^2,1)*qb0;
NNK1=find(abs(K_1(:,1)+i*K_1(:,2))<=0.8*NN*max(norm(b1m0),norm(b2m0))
);
NNK2=find(abs(K_2(:,1)+i*K_2(:,2))<=0.8*NN*max(norm(b1m0),norm(b2m0))
);

TKqb=t_vert/3*[1,1;1,1];
TKqtr=t_vert/3*[1,exp(-i*2*pi/3*(1+sqrt(3)*EExx*EExy+sqrt(3)*EEyy*EEx
y-EExy^2-EEyy^2));

exp(i*2*pi/3*(1+sqrt(3)*EExx*EExy+sqrt(3)*EEyy*EExy-EExy^2-EEyy^2)),1
];
TKqtl=t_vert/3*[1,exp(i*2*pi/3*(1-sqrt(3)*EExx*EExy-sqrt(3)*EEyy*EExy
-EExy^2-EEyy^2));

exp(-i*2*pi/3*(1-sqrt(3)*EExx*EExy-sqrt(3)*EEyy*EExy-EExy^2-EEyy^2)),
1];
T_Kqb=t_vert/3*[1,1;1,1];
T_Kqtr=t_vert/3*[1,exp(i*2*pi/3*(1+sqrt(3)*EExx*EExy+sqrt(3)*EEyy*EEx
y-EExy^2-EEyy^2));

exp(-i*2*pi/3*(1+sqrt(3)*EExx*EExy+sqrt(3)*EEyy*EExy-EExy^2-EEyy^2)),
1];
T_Kqtl=t_vert/3*[1,exp(-i*2*pi/3*(1-sqrt(3)*EExx*EExy-sqrt(3)*EEyy*EE
xy-EExy^2-EEyy^2));

exp(i*2*pi/3*(1-sqrt(3)*EExx*EExy-sqrt(3)*EEyy*EExy-EExy^2-EEyy^2)),1
];
qb=-4*pi/(3*sqrt(3)*d)*[EExx*cos(theta/2)+EExy*sin(theta/2),(2-EExx)*
sin(theta/2)+EExy*cos(theta/2)];
qtr=2*pi/(9*d)*[(sqrt(3)*EExx-3*EExy)*cos(theta/2)+(6-3*EEyy+sqrt(3)*
EExy)*sin(theta/2),-(3*EEyy-sqrt(3)*EExy)*cos(theta/2)+(2*sqrt(3)-sqr
t(3)*EExx+3*EExy)*sin(theta/2)];
```

```
qtl=2*pi/(9*d)*[(sqrt(3)*EExx+3*EExy)*cos(theta/2)-(6-3*EEyy-sqrt(3)*
EExy)*sin(theta/2),(3*EEyy+sqrt(3)*EExy)*cos(theta/2)+(2*sqrt(3)-sqrt
(3)*EExx-3*EExy)*sin(theta/2)];
b1m=2*pi/(3*d)*[-(sqrt(3)*EExx+EExy)*cos(theta/2)+(2-EEyy-sqrt(3)*EEx
y)*sin(theta/2),-(EEyy+sqrt(3)*EExy)*cos(theta/2)-(2*sqrt(3)-sqrt(3)*
EExx-EExy)*sin(theta/2)];
b2m=2*pi/(3*d)*[(sqrt(3)*EExx-EExy)*cos(theta/2)+(2-EEyy+sqrt(3)*EExy
)*sin(theta/2),-(EEyy-sqrt(3)*EExy)*cos(theta/2)+(2*sqrt(3)-sqrt(3)*E
Exx+EExy)*sin(theta/2)];
V_BZ=norm(cross([b1m,0],[b2m,0]));
ZZ=zeros(2);

[NN1,NN2]=meshgrid(-NN:NN);
NN1=reshape(NN1,(2*NN+1)^2,1);
NN2=reshape(NN2,(2*NN+1)^2,1);
K0=NN1*b1m+NN2*b2m;
K1=K0-ones((2*NN+1)^2,1)*qb;
K2=K0+ones((2*NN+1)^2,1)*qb;
K1=K1(NNK1,:);
K2=K2(NNK2,:);
NK1=length(K1);
NK2=length(K2);

KX_max=max([qb(1),qb(1)+qtr(1),qtr(1),-qb(1),qtl(1),qtl(1)+qb(1)]);
KX_min=min([qb(1),qb(1)+qtr(1),qtr(1),-qb(1),qtl(1),qtl(1)+qb(1)]);
KY_max=max([qb(2),qb(2)+qtr(2),qtr(2),-qb(2),qtl(2),qtl(2)+qb(2)]);
KY_min=min([qb(2),qb(2)+qtr(2),qtr(2),-qb(2),qtl(2),qtl(2)+qb(2)]);
Num=201;
[kx,ky]=meshgrid(1.2*linspace(KX_min,KX_max,Num),1.2*linspace(KY_min,
KY_max,Num));

for n1=1:length(K1)
    for n2=1:length(K2)
        if norm((K1(n1,:)-K2(n2,:))+qb)<1/2*norm(qb)
            HK_T(2*n1-1:2*n1,2*n2-1:2*n2)=TKqb;
            H_K_T(2*n1-1:2*n1,2*n2-1:2*n2)=T_Kqb';
        elseif norm((K1(n1,:)-K2(n2,:))-qb)<1/2*norm(qb)
            HK_T(2*n1-1:2*n1,2*n2-1:2*n2)=TKqb';
            H_K_T(2*n1-1:2*n1,2*n2-1:2*n2)=T_Kqb;
        elseif norm((K1(n1,:)-K2(n2,:))+qtr)<1/2*norm(qtr)
            HK_T(2*n1-1:2*n1,2*n2-1:2*n2)=TKqtr;
            H_K_T(2*n1-1:2*n1,2*n2-1:2*n2)=T_Kqtr';
        elseif norm((K1(n1,:)-K2(n2,:))-qtr)<1/2*norm(qtr)
            HK_T(2*n1-1:2*n1,2*n2-1:2*n2)=TKqtr';
```

```
            H_K_T(2*n1-1:2*n1,2*n2-1:2*n2)=T_Kqtr;
        elseif norm((K1(n1,:)-K2(n2,:))+qtl)<1/2*norm(qtl)
            HK_T(2*n1-1:2*n1,2*n2-1:2*n2)=TKqtl;
            H_K_T(2*n1-1:2*n1,2*n2-1:2*n2)=T_Kqtl';
        elseif norm((K1(n1,:)-K2(n2,:))-qtl)<1/2*norm(qtl)
            HK_T(2*n1-1:2*n1,2*n2-1:2*n2)=TKqtl';
            H_K_T(2*n1-1:2*n1,2*n2-1:2*n2)=T_Kqtl;
        else
            HK_T(2*n1-1:2*n1,2*n2-1:2*n2)=ZZ;
            H_K_T(2*n1-1:2*n1,2*n2-1:2*n2)=ZZ;
        end

        HK1_diff_x(2*n1-1:2*n1,2*n1-1:2*n1)=hbar_vf*[0,
((1+EExx)-i*EExy)*exp(i*theta/2);
            ((1+EExx)+i*EExy)*exp(-i*theta/2), 0];
        HK1_diff_y(2*n1-1:2*n1,2*n1-1:2*n1)=hbar_vf*[0,
(EExy-i*(1+EEyy))*exp(i*theta/2);
            (EExy+i*(1+EEyy))*exp(-i*theta/2), 0];

        H_K2_diff_x(2*n1-1:2*n1,2*n1-1:2*n1)=-hbar_vf*[0,
exp(i*theta/2);
            exp(-i*theta/2), 0];
        H_K2_diff_y(2*n1-1:2*n1,2*n1-1:2*n1)=-hbar_vf*[0,
i*exp(i*theta/2);
            -i*exp(-i*theta/2), 0];

        HK2_diff_x(2*n1-1:2*n1,2*n1-1:2*n1)=hbar_vf*[0,
exp(-i*theta/2);
            exp(i*theta/2), 0];
        HK2_diff_y(2*n1-1:2*n1,2*n1-1:2*n1)=hbar_vf*[0,
-i*exp(-i*theta/2);
            i*exp(i*theta/2), 0];

        H_K1_diff_x(2*n1-1:2*n1,2*n1-1:2*n1)=-hbar_vf*[0,
((1+EExx)+i*EExy)*exp(-i*theta/2);
            ((1+EExx)-i*EExy)*exp(i*theta/2), 0];
        H_K1_diff_y(2*n1-1:2*n1,2*n1-1:2*n1)=-hbar_vf*[0,
(EExy+i*(1+EEyy))*exp(-i*theta/2);
            (EExy-i*(1+EEyy))*exp(i*theta/2), 0];

    end
end
HK_diff_x=[HK1_diff_x,zeros(2*NK1,2*NK2);zeros(2*NK1,2*NK2),HK2_diff_
x];
```

```
HK_diff_y=[HK1_diff_y,zeros(2*NK1,2*NK2);zeros(2*NK1,2*NK2),HK2_diff_
y];
H_K_diff_x=[H_K2_diff_x,zeros(2*NK1,2*NK2);zeros(2*NK1,2*NK2),H_K1_di
ff_x];
H_K_diff_y=[H_K2_diff_y,zeros(2*NK1,2*NK2);zeros(2*NK1,2*NK2),H_K1_di
ff_y];

kkx=reshape(kx,Num^2,1);
kky=reshape(ky,Num^2,1);
kxv=[qb(1),qb(1)+qtr(1),qtr(1),-qb(1),qtl(1),qtl(1)+qb(1),qb(1)]';
kyv=[qb(2),qb(2)+qtr(2),qtr(2),-qb(2),qtl(2),qtl(2)+qb(2),qb(2)]';
[in,on]=inpolygon(kkx,kky,kxv,kyv);
kx_BZ=[kkx(in);kkx(on)];
ky_BZ=[kky(in);kky(on)];

for nn=1:length(kx_BZ)
    q=[kx_BZ(nn),ky_BZ(nn)];
    for n1=1:length(K1)

qK1=((1+EExx)*(q(1)-K1(n1,1)+A(1))+EExy*(q(2)-K1(n1,2)+A(2)))+i*(EExy
*(q(1)-K1(n1,1)+A(1))+(1+EEyy)*(q(2)-K1(n1,2)+A(2)));
        HK1(2*n1-1:2*n1,2*n1-1:2*n1)=hbar_vf*[0,
conj(qK1)*exp(i*theta/2);
            qK1*exp(-i*theta/2), 0]+MM1*[1,0;0,-1];
        q_K2=q(1)-K1(n1,1)+i*(q(2)-K1(n1,2));
        H_K2(2*n1-1:2*n1,2*n1-1:2*n1)=-hbar_vf*[0, q_K2*exp(i*theta/2);
            conj(q_K2)*exp(-i*theta/2), 0]+MM2*[1,0;0,-1];
    end
    for n1=1:length(K2)
        qK2=q(1)-K2(n1,1)+i*(q(2)-K2(n1,2));
        HK2(2*n1-1:2*n1,2*n1-1:2*n1)=hbar_vf*[0,
conj(qK2)*exp(-i*theta/2);
            qK2*exp(i*theta/2), 0]+MM2*[1,0;0,-1];

q_K1=((1+EExx)*(q(1)-K2(n1,1)-A(1))+EExy*(q(2)-K2(n1,2)-A(2)))+i*(EEx
y*(q(1)-K2(n1,1)-A(1))+(1+EEyy)*(q(2)-K2(n1,2)-A(2)));
        H_K1(2*n1-1:2*n1,2*n1-1:2*n1)=-hbar_vf*[0,
q_K1*exp(-i*theta/2);
            conj(q_K1)*exp(i*theta/2), 0]+MM1*[1,0;0,-1];
    end
    HK=[HK1,HK_T;HK_T',HK2];
    H_K=[H_K2,H_K_T;H_K_T',H_K1];
    [uK,vK]=eig(HK);
    [AK,BK]=sort(diag(vK),'ascend');
```

```
    %EK(:,nn)=AK(NK1+NK2:NK1+NK2+1);
    EK(:,nn)=AK(NK1+NK2+1);
    [u_K,v_K]=eig(H_K);
    [A_K,B_K]=sort(diag(v_K),'ascend');
    E_K(:,nn)=A_K(NK1+NK2+1);
    %E_K(:,nn)=A_K(NK1+NK2:NK1+NK2+1);

    WW=-1./(repmat(AK,[1,2*(NK1+NK2)])-repmat(AK,[1,2*(NK1+NK2)])');
    WW(isinf(WW))=0;

OrbitalK_mz=-1/2*imag((uK(:,BK(NK1+NK2+1))'*HK_diff_x*uK(:,BK).*WW(NK
1+NK2+1,:)).*transpose(uK(:,BK)'*HK_diff_y*uK(:,BK(NK1+NK2+1)))...

-(uK(:,BK(NK1+NK2+1))'*HK_diff_y*uK(:,BK).*WW(NK1+NK2+1,:)).*transpos
e(uK(:,BK)'*HK_diff_x*uK(:,BK(NK1+NK2+1))));


WW=-1./(repmat(A_K,[1,2*(NK1+NK2)])-repmat(A_K,[1,2*(NK1+NK2)])');
    WW(isinf(WW))=0;

Orbital_K_mz=-1/2*imag((u_K(:,B_K(NK1+NK2+1))'*H_K_diff_x*u_K(:,B_K).
*WW(NK1+NK2+1,:)).*transpose(u_K(:,B_K)'*H_K_diff_y*u_K(:,B_K(NK1+NK2
+1)))...

-(u_K(:,B_K(NK1+NK2+1))'*H_K_diff_y*u_K(:,B_K).*WW(NK1+NK2+1,:)).*tra
nspose(u_K(:,B_K)'*H_K_diff_x*u_K(:,B_K(NK1+NK2+1))));

    MK_z(:,nn)=e/hbar*0.1^2*sum(OrbitalK_mz,2)/mu_b;
    M_K_z(:,nn)=e/hbar*0.1^2*sum(Orbital_K_mz,2)/mu_b;

vKx(:,nn)=diag(uK(:,BK(NK1+NK2+1))'*HK_diff_x*uK(:,BK(NK1+NK2+1)));

vKy(:,nn)=diag(uK(:,BK(NK1+NK2+1))'*HK_diff_y*uK(:,BK(NK1+NK2+1)));

v_Kx(:,nn)=diag(u_K(:,BK(NK1+NK2+1))'*H_K_diff_x*u_K(:,BK(NK1+NK2+1))
);

v_Ky(:,nn)=diag(u_K(:,BK(NK1+NK2+1))'*H_K_diff_y*u_K(:,BK(NK1+NK2+1))
);
    %nn
end
MK_z_up=MK_z+1;%unit bohr magneton
M_K_z_up=M_K_z+1;% unit bohr magneton
MK_z_down=MK_z-1;
```

```
M_K_z_down=M_K_z-1;
gamma=1.21*1e-3;% unit eV
Ef=0.0012;% unit eV
Ef_p_up=Ef+gamma;
Ef_m_up=Ef-gamma;
dEK_up=1/4*beta0*(1-tanh(1/2*beta0*(EK-Ef_p_up)).^2);
F_EK_up=1./(1+exp(beta0*(EK-Ef_p_up)));
dE_K_up=1/4*beta0*(1-tanh(1/2*beta0*(E_K-Ef_m_up)).^2);
F_E_K_up=1./(1+exp(beta0*(E_K-Ef_m_up)));
chi=1/(2*pi)^2*sum(sum(MK_z_up.^2.*dEK_up+M_K_z_up.^2.*dE_K_up))*V_BZ
/length(kx_BZ)*1e2;%unit: bohr_magneton^2*eV^{-1}*nm^{-2}
Mag=1/(2*pi)^2*sum(sum(MK_z_down+M_K_z_down+MK_z_up.*F_EK_up+M_K_z_up
.*F_E_K_up))*V_BZ/length(kx_BZ)*1e2; % unit: bohr_magneton*nm^{-2}
Saturated_Mag=1/(2*pi)^2*sum(sum(MK_z_down+M_K_z_down+M_K_z_up))*V_BZ
/length(kx_BZ)*1e2; % unit: bohr_magneton*nm^{-2}
a0=1/(4*chi);%unit:bohr_magneton^{-2}*eV*nm^2
b0=1/(8*chi*Mag^2);%unit:bohr_magneton^{-4}*eV*nm^6
Bc=1/(3*sqrt(3))*Mag/(chi*5.788*1e-5);%unit:T
B0=1.5*linspace(-abs(Bc),Bc,3*1e2);
for nh=1:length(B0)
    M_root=roots([4*b0,0,-2*a0,-5.788*1e-5*B0(nh)]);
    if B0(nh)<0
        if real(M_root(2))==real(M_root(3))
            M0(1:2,nh)=[M_root(1);M_root(1)];
        else
            M0(1,nh)=M_root(2);
            M0(2,nh)=M_root(1);
        end
    else
        if real(M_root(2))==real(M_root(3))
            M0(1:2,nh)=[M_root(1);M_root(1)];
        else
            M0(1,nh)=M_root(1);
            M0(2,nh)=M_root(2);
        end
    end
end
figure,plot(B0,M0(1,:),'r-',B0,M0(2,:),'b-','LineWidth',2)
xlabel('B (T)');
ylabel('M (\mu_bnm^{-2})')
set(gca,'LineWidth',1,'FontSize',20,'Fontname','Times New Roman')

aa_zx=-1/(4*pi^2)*e/hbar*sum(sum(MK_z_up.*vKx.*dEK_up+M_K_z_up.*v_Kx.
*dE_K_up))*V_BZ/length(kx_BZ)*tau*10;%unit:mu_b/(V*nm)
```

```
aa_zy=-1/(4*pi^2)*e/hbar*sum(sum(MK_z.*vKy.*dEK_up+M_K_z.*v_Ky.*dE_K_
up))*V_BZ/length(kx_BZ)*tau*10;%unit:mu_b/(V*nm);
aa_zx=real(aa_zx);
aa_zy=real(aa_zy);
psi=pi/2;
Ec=2/3*sqrt(a0/(6*b0))*1/(aa_zx*cos(psi)+aa_zy*sin(psi));%unit: V/nm

E0=1.5*linspace(-abs(Ec),abs(Ec),3*1e2);
for ne=1:length(E0)

E_root=roots([4*b0,0,-2*a0,-2*a0*E0(ne)*(aa_zx*cos(psi)+aa_zy*sin(psi
))]);
    if E0(ne)<0
        if real(E_root(2))==real(E_root(3))
            M_E0(1:2,ne)=[E_root(1);E_root(1)];
        else
            M_E0(1,ne)=E_root(2);
            M_E0(2,ne)=E_root(1);
        end
    else
        if real(E_root(2))==real(E_root(3))
            M_E0(1:2,ne)=[E_root(1);E_root(1)];
        else
            M_E0(1,ne)=E_root(1);
            M_E0(2,ne)=E_root(2);
        end
    end
end
figure,plot(E0*1e9,M_E0(1,:),'m--',E0*1e9,M_E0(2,:),'m-','LineWidth',
2)
xlabel('E (V/m)','FontName','Times New
Roman','FontSize',36,'fontweight','b');
ylabel('M (\mu_bnm^{-2})','FontName','Times New
Roman','FontSize',36,'fontweight','b')
set(gca,'XTick',[-2,-1,0,1,2]*1e2)
set(gca,'YTick',[-1,-0.5,0,0.5,1]*1e-3)
set(gca,'LineWidth',1,'FontSize',28,'Fontname','Times New
Roman','fontweight','b')
```
